# Intervening for HIV prevention and mental health: a review of global literature

**DOI:** 10.1002/jia2.25710

**Published:** 2021-06-24

**Authors:** Pamela Y Collins, Jennifer Velloza, Tessa Concepcion, Linda Oseso, Lydia Chwastiak, Christopher G Kemp, Jane Simoni, Bradley H Wagenaar

**Affiliations:** ^1^ Department of Global Health University of Washington Seattle WA USA; ^2^ Department of Psychiatry and Behavioral Sciences University of Washington Seattle WA USA; ^3^ HIV Vaccine Trials Network, Vaccine and Infectious Disease Division Fred Hutch Seattle WA USA; ^4^ Department of Psychology University of Washington Seattle WA USA

**Keywords:** HIV prevention, mental health, prevention & control, severe mental illness

## Abstract

**Introduction:**

Numerous effective HIV prevention options exist, including behaviour change interventions, condom promotion and biomedical interventions, like voluntary medical male circumcision and pre‐exposure prophylaxis. However, populations at risk of HIV also face overlapping vulnerabilities to common mental disorders and severe mental illness. Mental health status can affect engagement in HIV risk behaviours and HIV prevention programmes. We conducted a narrative review of the literature on HIV prevention among key populations and other groups vulnerable to HIV infection to understand the relationship between mental health conditions and HIV prevention outcomes and summarize existing evidence on integrated approaches to HIV prevention and mental healthcare.

**Methods:**

We searched five databases for studies published from January 2015 to August 2020, focused on HIV prevention and mental health conditions among key populations and individuals with serious mental illness. Studies were included if they evaluated an HIV prevention intervention or assessed correlates of HIV risk reduction and included assessment of mental health conditions or a mental health intervention.

**Results and discussion:**

We identified 50 studies meeting our inclusion criteria, of which 26 were randomized controlled trials or other experimental designs of an HIV prevention intervention with or without a mental health component. Behaviour change interventions were the most common HIV prevention approach. A majority of studies recruited men who have sex with men and adolescents. Two studies provided distinct approaches to integrated HIV prevention and mental health service delivery. Overall, a majority of included studies showed that symptoms of mental disorder or distress are associated with HIV prevention outcomes (e.g. increased risky sexual behaviour, poor engagement in HIV prevention behaviours). In addition, several studies conducted among groups at high risk of poor mental health found that integrating a mental health component into a behaviour change intervention or linking mental health services to combination prevention activities significantly reduced risk behaviour and mental distress and improved access to mental healthcare.

**Conclusions:**

Evidence suggests that mental health conditions are associated with poorer HIV prevention outcomes, and tailored integrated approaches are urgently needed to address overlapping vulnerabilities among key populations and other individuals at risk.

## 1 Introduction

Despite an estimated 23% reduction in the global rate of human immunodeficiency virus (HIV) infection since 2010, dramatic disparities in the risk of HIV infection persist along the lines of entrenched social inequities. Over 60% of new HIV infections occur among key populations and their sexual partners, including gay men and other men who have sex with men (MSM), female sex workers (FSW), transgender women (TGW) (primarily), people who inject drugs (PWID), and prisoners and other incarcerated people [1]. In sub‐Saharan Africa, adolescent girls and young women (aged 15 to 24 years) account for 24% of new HIV infections despite representing only 10% of the population [1]. People with severe mental illnesses (SMI) are also over‐represented among new HIV infections [2, 3]. A diverse range of HIV prevention practices and technologies are effective, acceptable and increasingly available in many settings, including condom distribution and voluntary testing and counselling [4], universal test‐and‐treat [5], voluntary medical male circumcision (VMMC) [6], oral pre‐exposure prophylaxis (PrEP) [7], the dapivirine ring and other new, longer‐acting PrEP modalities [8] and combination prevention packages incorporating behavioural, biological and structural interventions [9, 10]. However, the over‐representation of key populations and other vulnerable groups among new HIV infections suggests that it is critical to improve their engagement with these effective HIV prevention strategies.

Among key populations and other vulnerable groups, the same inequities that drive HIV infection also increase the risk for mental health conditions [11]. Social exclusion and marginalization, poverty, violence and discrimination create cycles of vulnerability to both HIV and worsening mental health status [12]. A review of the mental health of sexual minorities reported elevated rates of depression, bipolar disorder, suicide attempts and drug use disorders across sexual orientation and genders [13]. Mental health conditions are disproportionately prevalent among incarcerated people; people in prisons are at increased risk of all‐cause mortality, suicide, self‐harm, violence and victimization [14].

Vulnerability to HIV and mental health conditions also intersect in adolescence, a sensitive period of neuropsychological and social development during which adolescents seek greater autonomy, takes risks and initiate sexual activity, with gender‐specific consequences [15, 16]. Globally, 1.7 million adolescents (ages 10 to 19) live with HIV, and nearly 60% of adolescents living with HIV are girls [17]. Most mental disorders that persist in adulthood begin in adolescence [18]. Young key populations have elevated rates of depression, suicidal ideation and intent and traumatic stress [19, 20]; meanwhile, adolescents with mental health conditions may be vulnerable to deficits in emotion regulation when making decisions about sex [21].

Lastly, people with SMI experience specific challenges related to living with symptoms that can be disabling, in addition to social, economic and gender‐specific vulnerabilities [22, 23, 24, 25, 26, 27]. These increase their risk of coercive sexual encounters, transactional sex, and sex with partners at high risk of HIV and unsafe drug use [28, 29, 30]. Notably, these groups are not mutually exclusive: individuals often have multiple marginalized identities leading to overlapping and intersecting vulnerability to both HIV and mental health conditions.

Few studies have examined HIV prevention and mental health among HIV‐negative key populations and vulnerable groups eligible for combination prevention, condom promotion, PrEP and other HIV prevention services. HIV prevention programmes providing behavioural counselling and PrEP interventions have found that young key populations have a high prevalence of depressive, anxiety and traumatic stress symptoms [19, 20, 31, 32]. Consequently, expert commentaries have called for integrated, culturally appropriate mental health and HIV prevention interventions to address the needs of people living with mental health conditions; these include social interventions, like cash transfer programmes, economic empowerment strategies and gender‐based violence services, alongside HIV prevention counselling, or PrEP delivery with psychotherapy and social support interventions to address mental health conditions [19, 20, 33, 34].

From 2014 to 2016, several reviews reported the evidence on the need for and results of HIV prevention interventions focusing on people with SMI [2, 35, 36, 37]. These reviews found that there was considerable heterogeneity in the outcomes of HIV prevention interventions in this population. Behavioural skills training and HIV risk reduction counselling were effective in reducing the number of sexual partners and condomless sex in some trials, but few studies demonstrated lasting effects post‐intervention [36].

A synthesis of more recent literature on the range of mental health conditions (from stress to SMI) is needed to understand the latest evidence on HIV prevention interventions and their outcomes for people managing or at risk for mental health conditions. We conducted a narrative review of global research on mental health and HIV prevention among adolescents and young women, key populations and people with SMI. Our primary aim was to understand how symptoms of mental health conditions influence HIV prevention intervention engagement and outcomes among these groups. We also reviewed evidence of integrated approaches to HIV prevention and the management of mental health conditions. We organize our findings around two questions: 1) what do we know about the relationship between mental health and HIV prevention or risk behaviours? 2) what do we know about interventions that address mental health in the context of HIV prevention?

## 2 Methods

We conducted a structured narrative review of the literature in peer‐reviewed journals to examine the relationships between HIV prevention, risk reduction and mental health conditions.

### 2.1 Search strategy

Two authors worked with a university informationist to select search terms and determine filters for five databases. We searched PubMed, Web of Science, CINAHL, PsychINFO and EMBASE for English‐language studies published between 1 January 2010 and 25 August 2020. We constructed search terms on HIV prevention; interventions, services or programmes; and mental disorders. We ran these base search terms with search terms for each of the three populations of interest: (1) adolescents (boys and girls) and young women, (2) people with SMI and (3) UNAIDS‐defined key populations (i.e. MSM, sex workers, transgender persons, PWID and prisoners and other incarcerated people as key population groups [38]). (See Additional File S1: Search Strategy.)

### 2.2 Inclusion and exclusion criteria

Studies were eligible if they met the following criteria: (1) assessed correlates of HIV risk reduction activities or evaluated an HIV prevention intervention; (2) included assessment of mental health conditions, cognitive processes or tailored an intervention for people with mental disorders. We included studies related to the use of structural and behavioural HIV risk reduction approaches as well as biomedical prevention interventions. We excluded qualitative studies. Given the scope of the literature on drug use and HIV prevention, which merits its own review, we eliminated studies focused on harm reduction and prevention or treatment of substance use disorders if there was no mental disorder data. We also eliminated studies exclusively focused on HIV treatment as prevention for this review. Although treatment as prevention studies have the ultimate goal of preventing HIV transmission, they tend to focus on populations living with HIV who may have distinct HIV care and mental health needs compared with at‐risk, HIV‐negative populations. Eligible studies reported the outcomes of HIV prevention trials, cross‐sectional and longitudinal analyses or quasi‐experimental studies.

### 2.3 Study selection and data extraction

We restricted our review of papers to January 2015 to August 2020. There were overlapping topical reviews of the literature prior to 2015 (e.g. for people with SMI). Given the scope of prevention research publications and the rapid evolution of HIV prevention science, the criterion of a 5‐year period of review targets current research most relevant to practitioners and researchers. However, if studies from this time period were sub‐studies, secondary analyses or otherwise related to a primary trial that met our inclusion criteria, we also added the primary trial to the dataset. We identified five such studies, three of which were published between 2010 and 2014. We included six studies published from 2015 to 2020 identified in the PrEP literature (e.g. references of other publications or adjacent studies in a search database) that met our criteria for inclusion. All studies were imported into Endnote (X9) to remove duplicates, book chapters, conference abstracts and theses. The resulting set was imported into Abstrackr (Tufts Evidence‐based Practice Center, beta version) for title and abstract review [39].

Authors (TC, JV, LO) conducted an initial screening of 20 abstracts to arrive at consensus on studies for inclusion and exclusion, followed by screening of all abstracts using Abstrackr Beta [39]. Full‐text manuscripts were assessed independently by authors (PC, TC, JV, LO, LC, JS). PC resolved disagreement in the process of study selection.

Authors used spreadsheet software to extract the following information: lead author, year of publication, country of study, sample size, study population, study design, study objective, HIV prevention approach, approach category, mental health component in intervention, HIV prevention outcome, mental health assessment used, and key HIV prevention/mental health finding. PC and JV conducted a second‐level review of all eligible manuscripts. Due to the heterogeneity of our sample in terms of intervention type, outcome and populations, we conducted a qualitative synthesis of study findings.

## 3 Results and Discussion

### 3.1 Study characteristics

**Figure 1:**
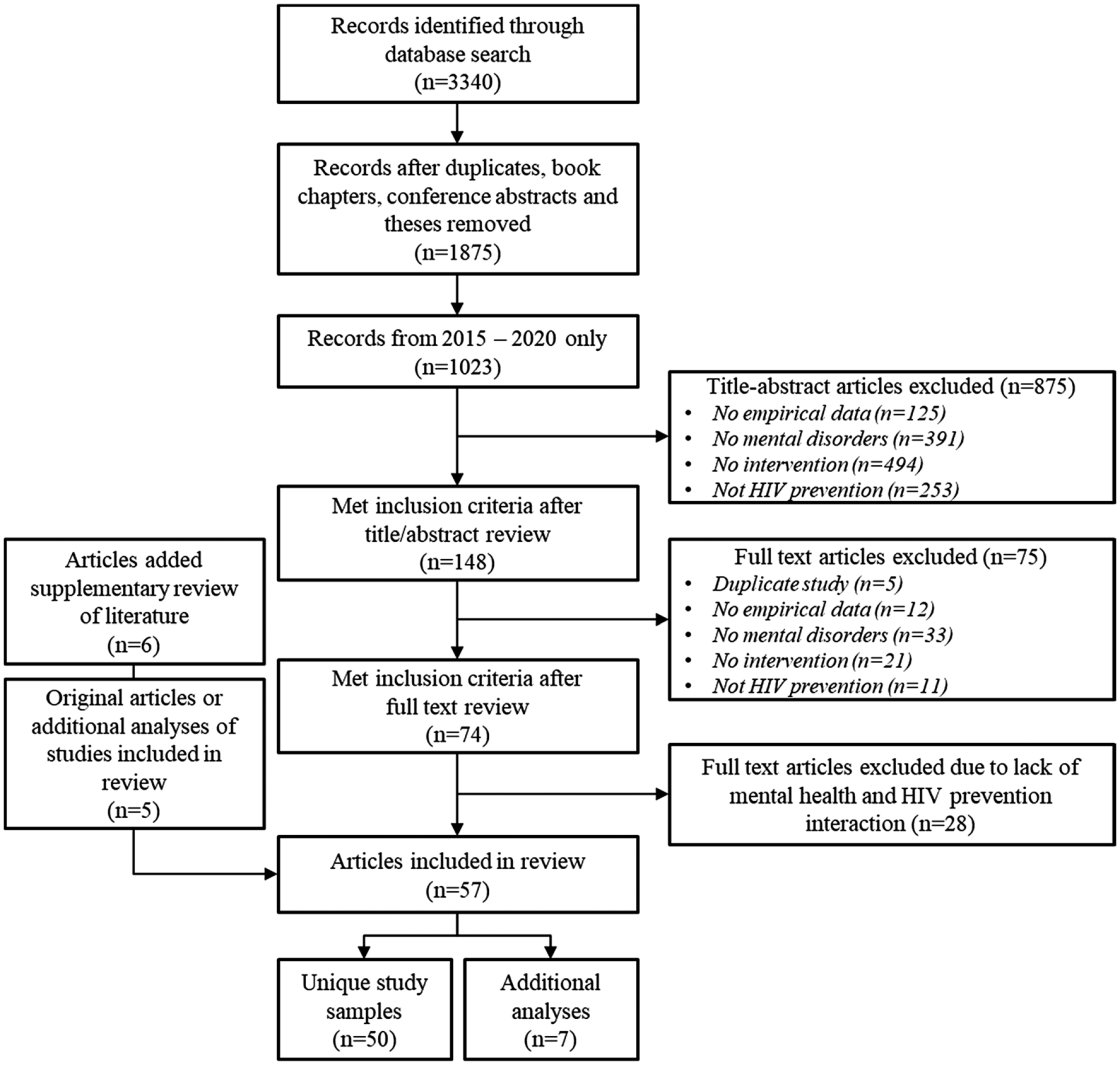
Flow diagram of search strategy.

A total of 3340 articles were identified through search criteria. After removing duplicates, book chapters, conference abstracts and theses, and restricting to 2015 to 2020 publication years, 1023 articles were eligible for the title/abstract screen. A total of 148 articles were full‐text reviewed and 46 were eligible to be included in this review. We added 11 articles after full‐text review, yielding a total of 57 articles in this review (Figure 1). We counted the full texts of multiple articles as one study if the same interventions were administered to the same study population or to a subset of the study population, reducing our total number of studies to 50.

Of the 50 studies we identified, 26 were randomized controlled trials or other experimental designs (e.g. quasi‐experimental designs with a pre‐ and post‐intervention period evaluation) testing the efficacy of an HIV prevention intervention with or without a mental health component, two were secondary analyses of randomized controlled trials, five were longitudinal studies examining risk reduction behaviour and 17 were cross‐sectional studies assessing the relationship of mental health status and HIV risk or prevention. The studies represent samples from 26 countries: United States (n = 23), Kenya (n = 5), South Africa (n = 4), Brazil (n = 5), Ecuador (n = 1), Peru (n = 3), Thailand (n = 3), Burkina Faso (n = 2), Belgium (n = 1), Canada (n = 2), China (n = 1), Colombia (n = 1), Côte d’Ivoire (n = 1), England (n = 2), Ethiopia (n = 1), India (n = 1), Jamaica (n = 1), Malaysia (n = 1), Mali (n = 1), Mexico (n = 1), Nepal (n = 1), Togo (n = 1), Uganda (n = 1), Vietnam (n = 1), Zambia (n = 1) and Zimbabwe (n = 1). The majority of studies recruited MSM (n = 19) and adolescents (n = 19). The remainder were transgender (n = 9), young women (n = 6), people with SMI (n = 3), FSW (n = 3), PWID (n = 3), mental health facilities (n = 1) and incarcerated persons (n = 1). The studies examined a range of HIV prevention approaches: 22 focused on HIV risk behaviour interventions, 12 on oral PrEP, 10 on condom use, 10 on voluntary counselling and testing, 6 on HIV knowledge/education, and 2 considered structural HIV prevention approaches such as free schooling and conditional cash transfers. Fewer studies examined biomedical interventions like treatment of sexually transmitted infections (STI) (n = 4), systems interventions (n = 1) and VMMC (n = 1). The studies measured mental health outcomes using screening assessments (n = 30), structured clinical interviews (diagnostic assessments) (n = 10) and other self‐report items (n = 32). We present study findings according to three population groups (adolescents and young women, key populations and people with SMI) and service integration, first describing trials and quasi‐experimental studies followed by non‐experimental designs for each group.

### 3.2 Adolescents and young women

Nineteen studies enrolled adolescents or young women and reported HIV prevention and mental health‐related factors as explanatory or outcome variables (Table 1). Depressive and anxiety symptoms were not associated with retention in a combined HIV risk reduction and alcohol and drug use reduction programme for homeless young adults [40].

**Table 1:** Adolescents (boys and girls) and young women – HIV prevention interventions with a mental health component or outcome (N = 19 unique studies)

Author year	Country	Study design	Study population	Intervention description	MH component	MH assessment	MH baseline prevalence and HIV/MH key findings
*Behavioural interventions*							
Brown 2013 [41]^a^	United States	RCT	377 youth (13 to 19 years)	3 arms with HIV prevention intervention focused on affect management, standard HIV knowledge and skills intervention, and general health promotion intervention control **Target**: HIV risk behaviour, affect dysregulation **Delivery**: Group / Schools **Facilitator**: Psychology postdoctoral fellows and bachelor‐level research assistants	Yes: affect management, informed by dialectic behaviour therapy techniques	Affect Dysregulation Scale, CIS, and C‐DISC‐IV	**MH‐related prevalence**: Affect dysregulation (14%) **MH outcome**: No effect on Affect Dysregulation Scale (ADS) score **HIV outcome**: Increased condom use affect management group after one month (F(2, 95) = 3.22, *p* = 0.04); HIV knowledge increased in both intervention groups (F(2, 314) = 7.89, *p* < 0.001)
Brown 2017 [42]							**MH‐related prevalence**: Affect dysregulation (14%) **MH outcome**: No effect on ADS or CIS scores **HIV outcome**: Increased condom use (aOR = 3.42, 95% CI: 1.10 to 10.63) and decreased sexual activity (aOR = 0.28, 95% CI: 0.08 to 0.96) in affect management intervention group at six months; HIV knowledge (F = 4.44, *p* = 0.04) and condom use attitudes (F = 3.86, *p* = 0.05) improved in both intervention groups.
Brown 2014 [48]^a^	United States	RCT	721 youth (13 to 18 years) and caregivers	3 arms with family‐based HIV prevention, adolescent‐only HIV prevention, or adolescent‐only health promotion **Target:** HIV risk behaviour, parental communication **Delivery:** Individual & Group / Inpatient and outpatient mental health settings **Facilitator:** One master or doctoral‐level clinician and one research assistant	Yes: skills training; framework that HIV behaviours are a function of psycho‐pathology and parenting	DISC, CIS‐13 item	**MH‐related prevalence**: Mental disorder (42%) **MH outcome**: Not reported (NR) **HIV outcome**: Increase in percentage of protected sex acts (RR = 59.04; 95% CI:16.50 to 82.23; *p* = 0.01) and decrease in unprotected sex acts (RR = 0.49; 95% CI: 0.28 to 0.86; *p* = 0.01) at three months
Barker 2019 [56]							**HIV outcome**: No difference in sexual behaviour 12 months; improved sex communication/parent monitoring at 12 months (d = .28 /d = .24)
Hadley 2015 [55]						SCL‐90R, GSI	**MH‐related prevalence**: Parental psychiatric impairment (32%) **HIV outcome**: Parents with elevated psychiatric symptoms had greater improvements on sexual communication at three months (t(211) = 2.09; *p* = 0.04; d = 0.17) and six months (t(315) = 2.23; *p* = 0.03; d = 0.18) and improved parental monitoring at three months (t(475) = 2.05; *p* = 0.04; d = 0.16).
Donenberg 2015 [49]	United States	Pre‐ and post‐test comparison design	54 juvenile offenders (13 to 17 years)	Social learning theory‐based comprehensive sex education programme **Target**: HIV risk behaviour; HIV knowledge, attitudes, beliefs; peer influence, partner relationships **Delivery**: Group / Community‐based Probation Service **Facilitator**: Trained study staff	Yes: emotion regulation	TAS and YSR – affect regulation	**MH**: NR **MH outcome**: No change in affect regulation **HIV outcome**: greater likelihood HIV counselling at three months (OR: 3.67, 95% CI: 1.66 to 8.11); improved HIV attitudes among girls (B = 2.25, *p* = .04); increased HIV knowledge (B = 1.74, *p* = 0.004)
Esposito‐Smythers 2017 [43]	United States	RCT	81 adolescents (13 to 17 years) and parents	Adjunctive cognitive‐behavioural family‐based alcohol, self‐harm, HIV prevention programme vs Assessment‐only control **Target**: HIV risk behaviour, suicidal self‐harm, substance use **Delivery**: Individual & Group / NR **Facilitator:** Master’s level interventionist	Yes: psycho‐education, cognitive behavioural skills training	CASA, DIS‐C, SITBI 2.0‐SF	**MH‐related prevalence**: Self‐harm (30.9%); risk of self‐harm (33.3%) **MH outcome**: Fewer self‐harm acts at 12 months (OR = 0.16, 95% CI: 0.03, 0.94) **HIV outcome**: greater refusal of sex to avoid an STI (OR = 4.87, 95% CI: 1.14, 20.9)
Houck 2016 [44]	United States	RCT	420 adolescents (12 to 14 years)	Emotion regulation intervention versus health promotion control **Target**: HIV risk behaviour, emotion regulation **Delivery**: Group / Schools **Facilitator**: Mental health clinician or trainee & research assistant	Yes: emotion regulation education and skills	DANVA‐2; DERS; ER Behaviours Scale	**MH‐related prevalence**: NR **MH outcome**: Significant difference on DANVA at six months, favouring ER condition (unstandardized estimate [b] = 2.91, 95% CI = 0.29 to 5.52) **HIV outcome**: Decreased likelihood of initiating vaginal sexual activity at 1 year (aHR = 0.58, 95% CI: 0.36 to 0.94, *p* = 0.01)
Jani 2016 [45]	Ethiopia	Pre‐ and Post‐ comparison design	576 female and 154 male adolescents (15 to 18 years)	Psychosocial counselling with individuals, groups, and creative therapies **Target**: HIV testing, HIV knowledge **Delivery**: Individual & Group / Service delivery organizations **Facilitator:** Study counsellors	Yes: problem‐solving therapy, group art therapy for emotional issues	YSR	**MH‐related prevalence**: Aggressive behaviour (23.0% females, 43.2% males); anxiety/depression (21.5% females, 47% males); any mental health problem (37.3% females, 80.8% males) **MH outcomes**: Decreased aggressive behaviour among females (aOR: 0.4, 95% CI: 0.25 to 0.65); decrease in reporting a mental health problem at three months (aOR: 0.5, 95% CI: 0.36 to 0.81) **HIV outcome**: Increased HIV knowledge (aOR: 1.6, 95% CI: 1.08 to 2.47) and HIV testing (aOR: 1.8, 95% CI: 1.13 to 2.97) for females; Increased HIV knowledge (aOR: 2.1, 95% CI: 1.10 to 3.94) and HIV testing (aOR: 7.3, 95% CI: 2.6 to 20.7) for males
Kendall 2020 [46]	United States	RCT	199 African American young females (14 to 18 years) and their mothers	Informed Motivated, Aware & Responsible about AIDS (IMARA) mother‐daughter dyad counselling intervention versus health promotion control **Target**: STI risk reduction **Delivery:** Group / Research site **Facilitator:** African American women with Bachelor’s or Master’s degree	Yes: exercises on externalizing and internalizing symptoms	YSR	**MH‐related prevalence**: NR **MH outcome**: No effect on internalizing or externalizing symptoms **HIV outcome**: Decreased incident STI infections through 12 months (Estimate = −.090, SE = 0.43, df = 195, t = −2.12, *p* = 0.035)
Logie 2015 [53]	Canada	Quasi‐experimental study	44 LBQ women (18+ years)	Group intervention targeting intrapersonal, community, and structural factors related to HIV **Target**: depression, STI knowledge, sexual stigma, HIV risk behaviour **Delivery:** Group / Weekend retreats **Facilitator:** Research coordinator and community facilitators	Yes: skills training, coping techniques	PHQ‐2	**MH‐related prevalence**: NR **MH outcome**: No effect on depression at six‐week follow‐up. **HIV outcomes**: Changes in sexual risk practices (β2 = −2.96, 95% CI ‐ 4.43, ‐ 1.50), barrier use self‐efficacy (β2 = 1.52, 95% CI 0.51, 2.53), STI knowledge (β2 = 4.41, 95% CI 3.52, 5.30), and sexual stigma (β2 = −2.62, 95% CI ‐ 3.48, ‐ 1.75) at six‐week post‐intervention.
Nall 2019 [58]	Kenya	Cross‐sectional	651 adolescents (13 to 24 years)	No intervention group **Outcome**: depressive symptoms, HTC	No	DASS‐21	**MH‐related prevalence**: NR **HIV outcome**: Depressive symptoms associated with lower intent to seek HIV testing (β = 0.019, χ^2^ = 3.72, *p* = 0.054)
Pearson 2019 [47]	United States	RCT	73 AI/AN women (18+ years)	Cognitive processing therapy intervention versus six‐week waitlist control **Target**: HIV risk behaviours, PTSD symptom severity, alcohol use **Delivery:** Individual / Behavioural health clinic **Facilitator:** Trained counsellors	Yes: cognitive‐processing therapy without a trauma narrative component	DSM‐IV symptom criteria, PTSD Symptom Scale Self‐report	**MH‐related prevalence**: PTSD diagnosis (65.8%) **MH outcome**: Reduction in PTSD through six weeks (d = 1.03, *p* < 0.001) **HIV outcome**: Reduction in HIV risk behaviours through six weeks (d = 1.02, *p* = 0.004)
Pedersen 2018 [40]	United States	RCT (group)	200 homeless young adults (18 to 25 years)	AWARE: Four‐session AOD and risky sex reduction programme **Target**: reducing AOD use behaviour, reducing risky sexual behaviours **Delivery**: Group / Drop‐in centres **Facilitator**: NR	No (Motivational interviewing for substance use and sexual risk reduction)	PHQ‐2 and GAD‐7	**MH‐related prevalence**: None reported **MH outcome**: No differences in HIV intervention retention by MH status (PHQ‐2, *p* = 0.654; GAD‐7, *p* = 0.573); retention significantly associated with homelessness severity (e.g. slept outside; estimate = −1.31, SE = 0.54, *p* = 0.015) HIV outcome : programme retention not related to sexual risk behaviour or severity of drug use
Puffer 2016 [50]	Kenya	Stepped wedge cluster RCT	237 adolescents (10 to 16 years) and 203 caregivers	*READY* family‐based intervention on economic empowerment, emotional support, and HIV education and prevention **Target**: family communication, HIV risk knowledge, self‐efficacy, and beliefs, HIV risk behaviour **Delivery:** Group / Churches **Facilitator:** Lay providers	Yes: cognitive behavioural approaches, mental health promotion	Subset of items from MASC‐10, CDI, and SDQ	**MH‐related prevalence**: None reported **MH outcome**: No effect on MH outcomes **HIV outcome**: Improved HIV risk knowledge (β = 0.03, 95% CI: 0.01, 0.31, *p* = 0.01), sex self‐efficacy (β = 0.41, 95% CI: 0.18, 0.64, *p* = 0.12), and high‐risk sex (β = −0.25, 95% CI: −1.31, −0.02, *p* = 0.12) at one month
Thurman 2018 [51]	South Africa	Quasi‐experimental pilot study	105 adolescents (13 to 17 years) and 95 female caregivers	*Let’s Talk* support group interventions with adolescents and their caregivers **Target**: HIV knowledge, behavioural skills, caregiver and adolescent mental health, parenting practices, HIV risk behaviour **Delivery:** Group / Community‐based organizations **Facilitator:** Trained facilitator	Yes: cognitive behavioural approaches, problem solving	DASS 21	**MH‐related prevalence**: NR **MH outcom**e: improved adolescent (Coeff. = −0.169, *p* = 0.004, Δ = −26.440%) and caregiver mental health (Coeff. = −0.223; *p* = 0.007; Δ = −22.468%) **HIV outcome**: improved adolescent HIV knowledge (Coeff. = 0.417, *p* = 0.008, Δ = 7.313%) and condom negotiation self‐efficacy (Coeff. = 0.501, *p* = 0.005, Δ = 11.710%),
Thurman 2020 [54]			64 female adolescents 13 to 17 years) and caregivers				**HIV outcome**: Caregivers' MH affected relationship quality, which affected parental sexual communication (R^2^ = 0.161 – indirect effects)
Zellner 2016 [52]	United States	Quasi‐experimental study	192 African American youth (18 to 24 years)	Colour It Real Programme, a culturally tailored HIV and substance use intervention **Target**: perceived stress, alcohol and drug use, HIV risk behaviour **Delivery:** Group / Colleges **Facilitator:** Trained staff	Yes: problem‐solving, skills training	Perceived Stress Scale	**MH‐related prevalence**: NR **MH outcome**: Decreased stress (t(70) = 2.38, *p* = 0.020) **HIV outcome**: Increased condom use (F = 4.43, *p* = 0.0360)
*Biomedical intervention*							
Luseno 2019 [61]	Kenya	Cross‐sectional	1939 young men (15 to 19 years)	No intervention group **Outcome**: depressive symptoms, VMMC	No	CES‐D‐R	**MH‐related prevalence**: Depressive symptoms (35%) **MH outcome**: Circumcised men had lower depressive symptoms (40.8% vs. 34.5%, χ^2^ = 4.40, *p* = 0.036)
Velloza 2020 [59]^a^	South Africa	Prospective cohort	174 women	**Exposure**: Depressive symptoms **Outcome**: PrEP adherence	No	CES‐D	**MH‐related prevalence**: Depressive symptoms (45.4%) **HIV outcome**: Depressive symptoms associated with PrEP adherence (aRR: 0.79, 95% CI: 0.63 to 0.99, *p* = 0.05)
*Social/structural intervention*							
Handa 2017 [57]	Kenya	Cluster RCT	1429 OVC (15 to 25 years)	Kenya Cash Transfer‐Orphans and Vulnerable Children (CT‐OVC) intervention providing household cash transfers to encourage fostering and retaining children **Target**: Depressive symptoms, sexual debut, schooling, socio‐economic status **Delivery**: Post offices	Yes	CESD‐10 and six‐item Hope Scale	**MH‐related prevalence**: Depressive symptoms (72.6%) **MH outcome**: Intervention improved mental health for males only (*p* = −0.031). No direct programme effects on mental health of girls. **HIV outcome**: Intervention reduced likelihood of sex by 9.4%. Schooling had a strong protective effect for girls (31% reduction in sexual debut probability). Psychosocial factors did not mediate relationship between intervention and sexual debut for girls. Among girls, fewer depressive symptoms and elevated hope reduced likelihood of sex debut by 10% (*p* < 0.10) and 8.6% (*p* < 0.05) respectively. No effect in boys.
Meinck 2019 [62]	South Africa	Prospective cohort	1498 adolescent girls (12 to 17 years)	**Exposure**: Access to free school **Outcome**: ACEs, depression, anxiety, HIV risk behaviour	No	UNICEF Scales for National‐Level Monitoring of Orphans and Other Vulnerable Children, CDI, RCMAS, MINI‐Kid	**MH‐related prevalence**: NR **MH outcome**: ACEs associated with internalizing behaviour (Effect: 0.808, *p* < 0.001) **HIV outcome**: ACEs associated with HIV risk behaviour (Effect: 0.145, *p* < 0.005). Free schooling weakened associations (Effect: 0.099, *p* < 0.05).
*Combination prevention*							
Sila 2020 [60]	Kenya	Cross‐sectional	470 AGYW	No intervention group **Outcome**: PrEP initiation, depressive symptoms, access to sexual and reproductive health services	No	CESD‐10	**MH‐related prevalence**: Depressive symptoms (13%) **HIV outcome**: Depressive symptoms associated with increased PrEP initiation (PR: 5.36, 95% CI: 2.62 to 10.95, *p* < 0.001)

ACEs, adverse childhood experiences; ADS, affect dysregulation scale; AGYW, adolescent girls and young women; AI/AN, American Indian/Alaska Native; CASA, the child and adolescent services assessment; CDI, children's depression inventory; C‐DISC‐IV, computerized diagnostic interview schedule for children; CES‐D, center for epidemiological studies depression scale; CES‐D‐R, center for epidemiological studies depression scale revised; CIS, Columbia Impairment Scale; DANVA, diagnostic analysis of nonverbal accuracy; DASS, depression, anxiety, and stress scale; DISC, diagnostic interview schedule for children 4.0; DSM‐IV, diagnostic and statistical manual of mental disorders, fourth edition; ER, emotion regulation; GAD, generalized anxiety disorder; GSI, global severity index; HIV, human immunodeficiency virus; LBQ, Lesbian Bisexual Queer; MASC, multi‐dimensional anxiety scale for children; MINI‐Kid, mini International psychiatric interview for children and adolescents; MH, mental health; NR, not reported; OVC, orphans and vulnerable children; PHQ, patient health questionnaire; PrEP, pre‐exposure prophylaxis; PTSD, post‐traumatic stress disorder; RCMAS, revised children's manifest anxiety scale; RCT, randomized controlled trial; SCL‐90R, the symptom checklist‐90 revised; SDQ, strengths and difficulties questionnaire; SITBI 2.0‐SF, self‐injurious thoughts and behaviour interview 2.0 ‐ Short Form; STI, sexually transmitted infections; TAS, toronto alexithymia scale; UNICEF, United Nations Children's Fund; YSR, youth self report.

^a^ Paper added after original search.

#### 3.2.1 Behavioural intervention trials and quasi experiments with a mental health component

Thirteen studies tested the efficacy of an HIV prevention intervention utilizing theory‐based skills building and reported both HIV and mental health outcomes among adolescents and young adults (Table 1). All studies demonstrated statistically significant effects on HIV prevention outcomes through follow‐up periods ranging from immediately post‐intervention to 12 months [41, 42, 43, 44, 45, 46, 47, 48, 49, 50, 51, 52].

Several studies clearly articulated behavioural or mental health symptom targets and integrated dialectical behaviour therapy techniques [41, 42], emotion regulation [44, 49], cognitive behavioural skills training and psychoeducation for self‐harm prevention [43] cognitive processing therapy [47], problem‐solving for stress reduction [52] and problem‐solving therapy and creative therapies for anxiety and aggressive behaviour [45]. Others identified externalizing/internalizing symptoms [46], emotional and mental health [53] as intervention components, but did not specify the interventional approach. Two studies integrated mental health promotion activities, including psychoeducation and cognitive‐behavioural skills‐building for parent‐youth communication, while introducing behavioural strategies for reducing sexual risk [50, 51]. The efficacy of the mental health components was mixed: six studies reported no effect of the mental health intervention on adolescents or young women [41, 42, 46, 48, 49, 50, 53].

Parental mental health outcomes were indirectly related to adolescent HIV risk behaviours in two behavioural intervention studies [48, 54, 55]. One showed that building a closer relationship to support improved communication about sexual health is partially contingent on supporting the mental health of the adolescent and the caregiver [54]. Another produced greater improvements in sexual communication and parental monitoring among parents with more psychiatric symptoms at the three‐month follow‐up [55]; adolescents reported significant reductions in sexual risk behaviours at three months [48], but not after 12 months [56].

#### 3.2.2 Structural intervention trials

One randomized controlled trial tested a cash transfer intervention, which significantly reduced depressive symptoms among young men only and delayed sexual debut among young men and women [57].

#### 3.2.3 The relationship of mental health to HIV prevention behaviours

Five studies assessed mental health as an explanatory variable for HIV risk or preventive behaviours (Table 1). Depressive symptoms were associated with a slightly lower intent to undergo HIV testing and counselling among Kenyan youth [58] and with lower PrEP adherence among South African young women, even after accounting for stigma and for PrEP optimism, i.e. belief in the protective effects of PrEP [59]. Depressive symptoms were associated with a greater likelihood to initiate PrEP, as were low social support and high perceived self‐efficacy to take medication daily, among Kenyan adolescent girls and young women [60].

Depressive symptoms were also measured in the context of VMMC service uptake and parental consent among circumcised and uncircumcised adolescents [61]. Circumcision without parental consent was associated with being an orphan, out of school, probable clinical depression (CES‐D score >16), and poorer quality of life. In addition, higher proportions of uncircumcised youth were depressed compared to the circumcised, possibly due to social pressure associated with VMMC campaigns or shaming of uncircumcised boys in high uptake communities.

A South African study of HIV risk, adverse childhood experiences (ACEs), adolescent mental health, and free schooling for adolescent girls, found that relationships between ACEs and HIV risk behaviour were mediated by internalizing and externalizing symptoms [62]. Free schooling was associated with fewer externalizing symptoms, suggesting that the mental health‐promoting effects of free education confer some protection against HIV risk.

### 3.3 Key populations

Twenty‐six studies enrolled members of key populations and reported HIV prevention outcomes and mental health‐related factors as explanatory or outcome variables (Table 2).

**Table 2:** Key populations— HIV prevention interventions with a mental health component or outcome (N = 26 unique studies)

Author year	Country	Study design	Study population	Intervention description	MH component	MH assessment	MH baseline prevalence and HIV/MH key findings
*Behavioural Intervention*							
Bao 2016 [74]	Vietnam	Cross‐sectional	204 TGW	No intervention group **Outcome**: HTC	No	Four‐item PTSD primary care screening tool.	**MH‐related prevalence**: None reported **HIV outcome**: PTSD associated with lower odds of HIV testing (aOR: 0.79, 95% CI: 0.64 to 0.96, *p* = 0.018)
Eke 2019 [63]	United States	Quasi‐experimental study	666 young black MSM	Mpowerment‐based community‐level intervention on psychosocial determinants of HIV risk behaviour **Target**: HIV risk behaviour, HTC, social diffusion **Delivery**: Group / Community **Facilitators**: Core groups and volunteers	Not specified	CES‐D‐9	**MH‐related prevalence**: None reported **MH outcome**: Significant favourable participation effect on reduction of depressive symptoms (3.9 vs. 4.7, F(1, 947) = 4.54, *p* = 0.03). **HIV outcome**: Community effects: favourable changes in social diffusion of safer sex messages (z (2477) = 2.92,*p* = 0.004) and comfort with being gay (z(2477) = 2.45, *p* = 0.01); Individual level: more social diffusion of safer sex messages (F(1926) = 6.58, *p* = 0.01); participants responded less favourably (*p* < 0.01) on sex in difficult situations and attitudes towards condom use
Hsu 2015 [82]	United States	Cross‐sectional	182 homeless MSM	No intervention group **Outcome**: Condom use	No	3‐item depression screen, PTSD Screen	**MH‐related prevalence**: Depression (52.2%); **HIV outcome**: Condom efficacy is an intervening variable (χ^2^ = 5.78, *p* < 0.001) on consistent condom use directly affected by depression (T = −3.53, *p* < 0.001)
Johnson 2015 [64]	United States	Pilot feasibility study	14 incarcerated women (18+ years)	Women’s Prison CoOp **Target**: unprotected vaginal or anal sex occasions, interpersonal violence, PTSD and depressive symptoms, alcohol and drug use, HIV risk behaviour, condom use **Delivery**: Small group & individual / Prison **Facilitators**: social worker, prison discharge planner, public health student	Yes: affect management psycho‐social education	THQ‐24, DTS‐17, and QIDS,	**MH‐related prevalence**: None reported **MH outcome**: PTSD symptoms (t = −2.27, df = 12, *p* = 0.04) and depressive symptoms (t = −2.87, df = 12, *p* = 0.01) decreased from baseline to 2 months post‐release follow‐up **HIV outcome**: Unprotected sex (t = −2.45, df = 12, *p* = 0.03) decreased from baseline to two months post‐release follow‐up
Logie 2018 [83]	Jamaica	Cross‐sectional	556 MSM	No intervention group **Outcome**: Condom use	No	PHQ‐2	**MH‐related prevalence**: None reported **HIV outcome**: Depression (ß = −0.035, *p* < 0.01) mediated relationship between stigma and condom use (ß = −0.169, *P* < 0.001)
Mimiaga 2012 [65]^a^	United States	Open phase pilot of intervention	19 MSM, PWID (18+ years)	Behavioural activation therapy and risk reduction counselling (BA‐RR) versus IMB skills change approach to sexual risk reduction control **Target**: HIV risk behaviour, drug use, anhedonia **Delivery**: Individual / Research centre **Facilitators**: Therapist	Yes: BA therapy	MADRS, Behavioural Activation Scale	**MH‐related prevalence**: None reported **MH outcome**: Statistically significant reductions in depressive symptoms were maintained (ß = −7.44, 95% CI: −13.04, −1.84, *p* = 0.013) **HIV outcome**: Intervention was associated with decreased unprotected anal intercourse at 3 (ß = −4.86; 95% CI: −7.48, ‐ 2.24; *p* = 0.0015) and six months (ß = −5.07; 95% CI: −7.85, ‐ 2.29; *p* = 0.0017)
Mimiaga 2019 [66]	United States	RCT	41 MSM, PWID (18+ years)		Yes: BA therapy	MADRS, Behavioural Activation Scale	**MH‐related prevalence**: None reported **MH outcome**: Intervention did not reduce depression (β = 2.47; 95% CI: −4.51, 9.45; *p* = 0.489) **HIV outcome**: Intervention reduced condomless anal sex at six months post intervention (β = −0.95; 95% CI: −1.44, −0.46; *p* < 0.0001
Newcomb 2017 [67]	United States	Pre‐test post‐test design	57 partners MSM	2GETHER couples‐based HIV prevention and relationship education consisted of four weekly, face‐to‐face sessions **Target**: HIV knowledge, relationship functioning, stress reduction **Delivery**: Group & individual couples / NR **Facilitators**: Bachelor’s and Master’s level clinical trainees, clinical psychologists; advanced clinical training not required	Yes: 2‐session psycho‐educational groups	PROMIS	**MH‐related prevalence**: None reported **MH outcome**: Intervention did not influence depression (t = 0.47, *p* = 0.641, *d* = 0.04) **HIV outcome**: Intervention showed decreases in HIV risk behaviour (*t* = −2.18, *p* = 0.032, *d* = 0.15)
O'Cleirigh 2019 [68]	United States	RCT	43 MSM (18+ years)	CBT for trauma and self‐care with HTC versus HTC alone control **Target**: HIV risk behaviour, condom use, and PTSD **Delivery**: Individual / Community health centre **Facilitators**: Clinical psychologists & pre‐ and post‐doctoral fellows in clinical psychology	Yes: CBT for trauma and self‐care	MINI‐6, DTS	**MH‐related prevalence**: None reported **MH outcome**: Intervention associated with reductions PTSD symptoms (γ_slope_ = −1.63, t(41) = −1.61, *p* = 0.11) through nine months **HIV outcome**: Intervention associated with reductions in condomless sex (γ_slope_ = −0.11, t (41) = 2.07, *p* = 0.04) through nine months
Ortblad 2020 [79]^a^	Uganda, Zambia	Secondary analysis from RCT	1925 FSW	3 study arms: (1) direct provision of an HIV self‐test from a peer educator; (2) facility collection of an HIV self‐test, or (3) referral to standard‐of‐care HIV testing services by a peer educator. **Target**: HTC, HIV knowledge **Delivery**: Group / Community **Facilitators**: Peer educators	No	PHQ‐9	**MH‐related prevalence**: Depressive symptoms: Uganda: 42.3%; Zambia 45.7%; Suicidal ideation: Uganda, 31.5%; Zambia, 56.7% **MH outcome**: Knowledge of any HIV status associate with reductions in severity of depressive symptoms in both sites. Knowledge of HIV‐positive status associated with a 1.01‐point decrease in depressive symptoms in Uganda (95% CI: −1.82, −0.20, *P* = 0.02) and 1.98‐point decrease in depressive symptoms in Zambia (95% CI: −3.09, −0.88, *P* = 0.001). Knowledge of any HIV status associated with reduced prevalence of likely depression in Zambia
Reisner 2016 [69]	United States	Open phase pilot of intervention	17 young transgender MSM (18 to 29 years)	LifeSkills for Men (LS4 M) uses modified social ecological model of HIV risk to conceptualize the multiple contexts and dimensions of sexual risk behaviours for young transgender MSM. **Target**: HIV risk behaviour **Delivery**: Small group / NR **Facilitators**: Study team and transgender MSM community member	No	BSI	**MH‐related prevalence**: None reported **MH outcome**: Trends suggest that intervention may reduce psychological distress: BSI 19.4 baseline to 17.5 at four months (*t* = −1.66, df = 16, *p* = 0.28), not significant. **HIV outcome**: increase in condom self‐efficacy (t = 2.11, df = 16, *p* = 0.05) at four months; other changes not significant.
Rutledge 2018 [77]	Malaysia	Cross‐sectional	199 TGW	No intervention group **Outcome**: HTC	No	HRQoL SF‐12; self‐report	**MH‐related prevalence**: High MH functioning (49.2%) and previous depression diagnosis (7.0%) **HIV outcome**: High MH functioning associated with HIV testing (aOR: 2.27, 95% CI: 1.04 to 4.96, *p* = 0.041), previous depression diagnosis associated with HIV testing (aOR: 6.16, 95% CI: 1.56 to 24.24, *p* = 0.010).
Shrestha 2017 [75]	Nepal	Cross‐sectional survey	1010 FSW, MSM, TG	No intervention group **Outcome**: HTC	No	CES‐D and SI	**MH‐related prevalence**: None reported **HIV outcome**: Depression associated with not using HTC for FSW (aPR 1.4, 95% CI 1.1 to 1.6).
Tobin 2017 [70]	United States	RCT	315 PWID (18+ years)	Fiveweeks, 10 session CBT and HIV integrated intervention **Target**: CBT skills, HIV risk behaviour, condom use **Delivery**: Group / NR **Facilitators**: Lay facilitators w/ high school education/equivalent & 10 years group facilitation experience	Yes: Integrated CBT and HIV prevention intervention	CES‐D	**MH‐related prevalence**: Depressive symptoms (100%) **MH outcome**: Intervention reduced depressive symptoms at 12 months (OR: −2.83, 95% CI: −5.28, −0.38, *p* < 0.05); significant reductions in CES‐D over baseline at 6 months and 12 months, though no difference from control group at six months. **HIV outcome**: Intervention increased condom use with non‐main partner at six months (OR: 1.99, 95% CI: 1.03, 3.83, *p* < 0.05)
Wei 2016 [76]	China	Cross‐sectional survey	523 MSM	No intervention group **Outcome**: HTC	No	CES‐D‐10	**MH‐related prevalence**: None reported **HIV outcome**: Depression/stigma associated with lower odds of HIV testing (aOR: 0.96, 95% CI: 0.92 to 0.99; and aOR: 0.91, 95% CI: 0.84 to 0.99 respectively). Relationship between homophobia and testing mediated 35.0% through depression (OR_NIE_: 0.96, 95% CI: 0.93 to 0.98).
*Biomedical*							
Edeza (2019)[116]^a^	Mexico, Brazil, Colombia	Online survey	22,698 MSM	No intervention group **Outcome**: PrEP awareness, PrEP use and interest in PrEP trial participation	No	CES‐D‐10	**MH‐related prevalence**: Depressive symptoms (72.7%) **HIV outcome**: 10.4% were aware of PrEP; Transactional sex and CAS were associated with increased PrEP awareness (aOR: 1.29, 95% CI: 1.05–1.59, *p* < .001 and aOR: 1.22, 95% CI: 1.11–1.34, *p* < 0.001 respectively) and PrEP trial interest (aOR: 1.45, 95% CI: 1.25–1.71, *p* < 0.001 and aOR: 1.74, 95% CI: 1.57–1.95, *p* < 0.001 respectively)
Goodman 2016 [78]	Burkina Faso	Cross‐sectional	672 MSM	No intervention group **Outcome**: STI testing	No	Questions on depressive symptoms and SI	**MH‐related prevalence**: Depressive symptoms (36%) **HIV outcome**: Ever having depression was associated with STI testing (aOR: 1.49, 95% CI: 1.01 to 2.20, *p* < 0.05)
Grant 2010 [73]^a^	United States, South Africa, Thailand, Peru, Ecuador, Brazil	RCT	2499 MSM, TGW	Pre‐exposure Prophylaxis Initiative (iPrEx) trial **Target**: Safety and efficacy of PrEP **Delivery**: Individual / Clinic **Provider**: Study investigators	No	Depression‐related AEs, suicide attempts	**MH‐related prevalence**: Depression‐related AEs (69%), suicide attempts (5%) **MH outcome**: No difference in depression‐related AEs by PrEP (3% of patients) versus placebo arms (4% of patients)
Defechereux 2016 [71]						CES‐D and 4‐item suicidal ideation screen	**MH‐related prevalence**: Suicidal ideation: 8%, n = 36 suicide attempts **MH outcome**: Predictors of depression: forced anal sex debut (Coeff: 3.23, 95% CI: 1.24 to 5.23), TGW (Coeff: 1.22, 95% CI: 0.51 to 2.40), younger age (Coeff: 1.25, 9% CI: −0.07 to 2.57). Predictors of SI: African American (OR: 2.15, 95% CI: 1.09–4.26); participants reporting forced anal sex debut (OR: 2.2, 95% CI: 1.31 to 5.53). **HIV outcome**: Non‐condom receptive anal intercourse associated with higher CES‐D (OR: 1.46, 95% CI: 1.09 to 1.94, Wald test for linearity: *p* = 0.012)
Mehrotra 2016 [72]^a^		Nested case‐control	334 MSM and TGW			CES‐D	**MH‐related prevalence**: Depressive symptoms (28%) **HIV outcome**: Depressive symptoms were moderately associated with PrEP nonadherence (OR: 0.41, 95% CI: 0.22, 0.77), results differed between MSM and TGW.
Miltz 2019a [86]^a^	England	Open label PrEP RCT	554 GBMSM	The PROUD clinical trial evaluated the efficacy of PrEP against HIV acquisition among GBMSM in England **Target**: PrEP benefit **Delivery:** Individual / Clinic **Facilitators:** NR	No	PHQ‐9	**MH‐related prevalence:** Depression (9.1%) **MH outcome:** Depression increased significantly from baseline (9.1%) to the 12‐month (14.4%) and 24‐month (14.4%) follow‐up. IPV (aPR 2.57, 95% CI: 1.71 to 3.86), internalized homophobia (aPR 1.91, 95% CI: 1.29 to 2.83) and concealment of sexual identity (aPR 1.75, 95% CI: 1.16 to 2.65) were strongly associated with depression
Miltz 2019b [85]^a^			436 GBMSM				**MH outcome:** Depressive symptom prevalence was higher in men who reported combined lifetime IPV victimization/ perpetration (aPR: 3.87, 95% CI: 2.43 to 6.16, *p* < 0.001) **HIV outcome:** Depressive symptoms were not associated with sexual risk behaviours in unadjusted or adjusted analysis; lifetime and past IPV were not associated with sexual risk behaviours
Nostlinger 2020 [84]	Belgium	Prospective cohort	200 MSM	**Exposure**: PrEP **Outcome**: Depressive symptoms **Delivery**: Individual / Clinic **Facilitator:** NR	No	PHQ‐9	**MH‐related prevalence**: Depressive symptoms (12%) **HIV outcome**: Interaction of drug use and depression on sexual risk at baseline (Estimate: 0.097, *p* = 0.039); after PrEP introduced, no interaction seen at month 9 and 18
Pagkas‐Bather 2020 [89]	United States	Cross‐sectional survey	95 MSM	No intervention group **Outcome**: Peer navigator acceptability and PrEP uptake	No	PHQ‐9	**MH‐related prevalence**: None reported **HIV outcome**: High PHQ‐9 associated with higher peer navigator acceptability (ß = 0.04, 95% CI: 0.01 to 0.07, *p* = 0.01), but the association was not significant in adjusted models
Pasipanodya 2018 [88]	United States	Secondary analysis from RCT	181 MSM	Text‐messaging versus standard of care **Target**: PrEP adherence **Delivery**: Individual / Clinic **Facilitators**: NR	No	PHQ‐9	**MH‐related prevalence**: Depressive symptoms (48.6%) **HIV outcome**: Depression symptoms associated with lower PrEP adherence (OR:1.111, 95% CI: 1.005 to 1.227, *p* < 0.05)
Shuper 2020 [90]^a^	Canada	Cross‐sectional	141 GBMSM	No intervention group **Outcome**: PrEP non‐adherence	No	CES‐D	**MH‐related prevalence**: Depressive symptoms (23.7%); Harmful/hazardous drinking (31.9%); moderate/high risk substance use (43.3%) **HIV outcome**: Depression was not associated with nonadherence
Young 2020 [87]	United States	Cross‐sectional	31 young MSM/ TGW of colour	No intervention group **Outcome**: PrEP adherence	No	PHQ‐9, GAD‐7, and ACEs	**MH‐related prevalence**: NR **HIV outcome**: Anxiety (80.7% vs. 92.7%, *p* = 0.04) and trauma experiences (84.5% vs. 95.7%, *p* = 0.05) associated with PrEP non‐adherence; depression not associated (*p* = 0.28)
*Combination prevention*							
Chabata 2020 [81]	Zimbabwe	Cohort study	2431 young women who sell sex (18 to 24 years)	DREAMS programme: biomedical, social and economic interventions **Target**: PrEP, HIV risk behaviour, condom use **Delivery**: Community / NR **Facilitators**: Community, faith‐based and non‐governmental organizations	No	SSQ‐14	**MH‐related prevalence**: CMD (33.6%) **HIV outcome**: Lower odds of condom use for those at risk of CMD in past week (aOR = 0.76; 95% CI: 0.60 to 0.97; *p* = 0.029)
Coulaud 2019 [80]	Mali, Cote d’Ivoire, Burkina Faso and Togo	Longitudinal study	621 MSM	Community‐based cohort providing quarterly HIV testing and counselling **Target**: HIV risk behaviour, HTC, STI treatment, PrEP, condom use **Delivery**: Individual / Clinic **Facilitators**: Physicians & peer educators	No	PHQ‐9	**MH‐related prevalence**: NR **HIV outcome**: High level of depressive symptoms was associated with inconsistent condom use during receptive anal sex with male partners of unknown HIV status (OR: 1.06, 95% CI: 1.00 to 1.13, *p* = 0.049)

ACEs, adverse childhood experiences; AE’s, adverse events; BA, Behavioural Activation; BSI, brief symptom inventory; CBT, cognitive‐based therapy; CES‐D, center for epidemiological studies depression scale; CMD, common mental disorders; DTS, davidson trauma scale; FSW, female sex worker; GAD, Generalized Anxiety Disorder; GBMSM, gay and bisexual men who have sex with men; HIV, Human Immunodeficiency Virus; HRQoL SF‐12, Health‐Related Quality of Life SF‐12; HTC, hiv testing and counselling; IMB, Information‐motivation‐behavioural; IPV, intimate partner violence; MADRS, montgomery‐asberg depression rating scale; MH, mental health; MINI, mini international neuropsychiatric interview; MSM, men who have sex with men; NR, not reported; PHQ, Patient Health Questionnaire; PrEP, Pre‐Exposure Prophylaxis; PROMIS, patient‐reported outcomes measurement information system depression—short form 8b; PTSD, post‐traumatic stress disorder; PWID, Persons Who Inject Drugs; Qids, Quick Inventory Of Depressive Symptomatology; RCT, randomized control trial; SI, suicidal intent; SSQ, shona symptom questionnaire; STI, sexually transmitted infections; TG, transgender person; TGW, transgender women; THQ, trauma history questionnaire.

^a^ Paper added after original search.

#### 3.3.1 Behavioural intervention trials and quasi‐experiments

Eight studies reported the efficacy of a theory‐based intervention for HIV risk reduction [63, 64, 65, 66, 67, 68, 69, 70]. Most reported favourable changes in risk reduction. One pilot intervention’s results did not achieve significance [69], and a community‐level intervention yielded positive and negative outcomes [63] (Table 2). Of these studies, six integrated mental health components including psychoeducation for depression and post‐traumatic stress disorder (PTSD) and affect management to reduce dissociation [64], behavioural activation for methamphetamine‐related anhedonia [65, 66], psychoeducation and cognitive‐behavioural and acceptance‐based coping with stressors [67], cognitive behavioural therapy (CBT) for trauma [68], and CBT for depression [70]. With the exception of one study that did not describe the mental health component [63], interventions with significant reductions in mental disorder symptoms (depression, anhedonia, PTSD) utilized CBT, behavioural activation and affect management with psychoeducation. All interventions were delivered in group formats.

#### 3.3.2 Biomedical intervention trials

Analysis of a PrEP safety trial among MSM and TGW revealed a high prevalence of moderate depression across participants in both study arms, but no difference in depression‐related adverse events or reports of suicide attempts and self‐harm between participants in the PrEP and placebo arms [71]. A sub‐group analysis showed possible severe depression for MSM and possible moderate depression for TGW were associated with reduced PrEP adherence, but were infrequent [72]. The authors emphasized the importance of ensuring access to PrEP for people with depressive symptoms [71, 72, 73].

#### 3.3.3 The relationship of mental health to HIV prevention behaviours

Eighteen studies examined mental health as a correlate of HIV risk reduction in studies of HIV or STI testing and counselling, condom use, and awareness of, adherence to or engagement with PrEP (Table 2).

Of these, seven examined the relationship of mental health variables to HIV or STI testing in cohorts and community samples (Table 2). In four studies, *fewer symptoms* of a mental disorder were associated with HIV or STI testing, and greater severity of symptoms was associated with less testing [74, 75, 76]. HIV testing among TGW in Malaysia was associated with higher current scores of mental health functioning as well as having a *previous* diagnosis of depression [77]. In contrast, ever having a depressed mood for more than two weeks was independently associated with having an STI test in the past month among MSM in Burkina Faso [78]. Notably, learning one’s HIV status, whether positive or negative, was associated with significant reductions in the severity of depressive symptoms among Ugandan and Zambian FSW populations and was not associated with suicidal ideation [79].

Several studies demonstrated that poorer mental health (especially severe depression, depression and PTSD, or anxiety) was associated with less consistent condom use or less perceived condom self‐efficacy among MSM [80], young women who sold sex [81], and homeless men who also used drugs and traded sex [82]. Condom use self‐efficacy and depression were partial mediators of the relationship between sexual stigma and inconsistent condom use among MSM [83]. MSM on PrEP who reported poly‐drug use and depression were significantly more likely to report receptive condomless anal intercourse than those who only reported poly‐drug use or only reported depression at baseline assessment [84]. Conversely, among gay and bisexual MSM receiving PrEP in the United Kingdom, despite increased depressive symptoms over time, neither depressive symptoms nor interpersonal violence were associated with sexual risk behaviours [85, 86].

Two studies showed that distinct categories of mental disorder symptoms were associated with lower adherence to PrEP: anxiety symptoms and a history of childhood trauma [87], baseline depression and substance use among MSM in a PrEP adherence trial [88]. A third study reported higher depression scores were significantly related to greater acceptability of peer navigation to assist with PrEP engagement among Black and Latinx MSM [89]. A fourth study found no relationship of depressive symptoms to PrEP adherence, but harmful alcohol use and moderate/high‐risk cocaine use predicted nonadherence [90].

### 3.4 People with severe mental illness

Three studies enrolled people with SMI in a clinical trial or cross‐sectional surveys.

#### 3.4.1 HIV prevention interventions tailored for adults with severe mental disorders

A randomized controlled trial tested a sexual health promotion intervention among adults with SMI [91] (Table 3). Participants attending a three‐session theory‐based group intervention reported fewer episodes of unprotected sex acts through six months [91]. This study examined sexual health, broadly, as an area of attention for people with SMI.

**Table 3:** People with a serious mental illness—HIV prevention interventions with a mental health component or outcome (N = 3)

Author year	Country	Study design	Study population	Intervention description	MH component	MH assessment	MH baseline prevalence and HIV/MH key findings
*Behavioural intervention*							
Hughes 2019 [91]	England	RCT	72 adults (18+ years) with SMI	Sexual health promotion intervention (RESPECT) versus usual care alone **Target**: HIV risk behaviour and condom use **Delivery**: Individual / Community mental health services **Facilitator**: Mental health worker	No	Diagnosis through EMR and self‐referral	**MH‐related prevalence**: None reported **HIV outcome**: Intervention was associated with decrease in the number of unprotected sex acts through three months (4% reduction vs. 9.7% increase in control).
Pinho 2020 [92]	Brazil	Cross‐sectional	467 adults with SMI	No intervention group **Outcome**: Condom use	No	BPRS	**MH‐related prevalence**: None reported **HIV outcome**: Greater negative symptom severity was related to worse condom‐self efficacy (F(1, 452) = 3.75, *p* < 0.01) (β = −0.05, t = −2.69, *p* < 0.01), while greater activation symptom severity (e.g. elated mood) was related to better condom self‐efficacy (β = 0.04, t = 2.13, *p* = 0.03)
Wainberg 2018 [93]	Brazil	Cross‐sectional	641 adults with SMI	No intervention group **Outcome**: Participation in HIV risk reduction programme.	No	MINI PLUS	**MH‐related prevalence**: None reported **HIV outcome**: Only 9% of adults with SMI participated in a HIV risk reduction programme

BPRS, the expanded brief psychiatric rating scale; EMR, electronic medical record; HIV, human immunodeficiency virus; MINI PLUS, mini international neuropsychiatric interview – PLUS; MH, mental health; NR, not reported; RCT, randomized control trial; SCID, structured clinical interview for DSM‐IV diagnosis; SMI, serious mental illness.

#### 3.4.2 The relationship of mental health to HIV prevention behaviours

Two Brazilian studies examined people with SMI and HIV risk correlates (Table 3). One study showed that psychiatric symptom clusters had differential effects on condom self‐efficacy: people with more severe negative symptoms (e.g. blunted affect, emotional withdrawal) were less likely to perceive themselves as capable of using condoms, condom negotiation, and/or condom acquisition [92]. In a second study, three‐fourths of patients in eight public psychiatry clinics reported unprotected sex, but only 9% had participated in the clinics’ risk‐reduction programmes [93]. Participation was significantly associated with ethnicity, higher HIV knowledge, and receiving HIV testing in the past three months.

### 3.5 HIV prevention and mental health service integration

Two studies in our sample described distinct approaches to HIV prevention and mental health service integration (Table 4). One assessed the outcomes of the Pehchan programme, a community‐level intervention, which linked transgender persons to comprehensive community‐based services providing combination prevention to transgender persons in India [94]. The programme yielded significant increases in testing referrals, HIV education reach, and access to mental health support through referrals to psychological services.

**Table 4:** Integrated services (N = 2)

Author year	Country	Study design	Study population	Intervention focus	MH component	MH assessment	MH baseline prevalence and HIV/MH key findings
*Combination prevention*							
Shaikh 2016 [94]	India	Pre‐post non‐randomized design	268 TG	Pehchan programme supports CBOs in technical capacity, linkage to care and prevention interventions, and packages of care for supportive environments for transgender communities through legal, social, mental health, and psychosocial services **Target**: HIV, health, legal and social protection services **Delivery**: CBO / Community **Implementor**: India HIV/AIDS Alliance	No	Questions on access to psychological services	**MH‐related prevalence**: None reported **MH outcome**: Access to psychological services increased (+33.0%, *p* < 0.001) **HIV outcome**: Increase in access to HIV education (+20%, *p* < 0.001), referral for HIV testing (+33.7%, *p* < 0.001), access to condoms (+12.5%, *p* < 0.001) and condom use with regular (+18.1%, *p* < 0.001) and casual (+8.1%, *p* < 0.001) partners
McKinnon 2020 [95]	United States	Survey	132 outpatient mental healthcare agencies	No intervention group **Outcome**: Condom use, HTC	No	Licensed outpatient mental healthcare programmes	**MH‐related prevalence**: None reported **HIV outcome**: MH providers report decreases in condom distribution (*p* < 0.001) and increases in on‐site HIV testing (*p* < 0.001).

CBO, community‐based organization; HIV, human immunodeficiency virus; HTC, HIV testing and counselling; MH, mental health; STI, sexually transmitted infections; TG, transgender person.

A survey of mental health programme directors in New York State showed that a majority of programmes treated people known to have HIV, assessed HIV risk, and provided HIV educational materials, and just over half referred people for HIV testing [95]. Between 20% and 32% of programmes offered services related to *End the Epidemic* activities in the state (e.g. HIV testing, PrEP education and PrEP prescriptions). Compared to past surveys, fewer mental health programme directors reported integration of HIV services and psychiatric services, and fewer identified themselves as fully integrated in 2017 compared to 2004, despite more programmes reporting larger caseloads of people with HIV or AIDS.

### 3.6 Summary: Interventions that address mental health in the context of HIV prevention

Thirteen studies in our sample describe HIV prevention interventions (11 at the individual level, one social intervention, and one community systems intervention) with an embedded or linked mental health component(s) that reduced HIV risk behaviours and produced a more favourable mental health outcome (Table 5). The majority of these interventions or services occurred in the community, and one occurred in a prison.

**Table 5:** RCTs and comparison design studies that include both MH and HIV components (n = 21 unique study samples; n = 25 unique analyses)

	MH intervention components (by unique study samples)				Outcomes (by unique analysis)	
	Behavioural interventions incorporating parental communication skills	Non‐specific management of psychological processes or distress^a^	Disorder‐specific management^b^	Other interventions	Any favourable MH outcome	Any favourable HIV prevention outcome
AYW (n = 13 unique study samples; n = 17 unique analyses)	Brown 2014 [48]/ Barker 2019 [56]/ Hadley 2015 [55] Esposito‐Smythers 2017 [43] Kendall 2020 [46] Puffer 2016 [50] Thurman 2018 [51]/ Thurman 2020 [54]	Brown 2013 [41]/ Brown 2017 [42] Donenberg 2015 [49] Houck 2016 [44] Kendall 2020 [46] Zellner 2016 [52]	Esposito‐Smythers 2017 [43] Jani 2016 [45] Logie 2015 [53] Pearson 2019 [47]	Social/Structural Handa 2017 [57]	Esposito‐Smythers 2017 [43] Houck 2016 [44] Jani 2016 [45] Pearson 2019 [47] Thurman 2018 [51] Zellner 2016 [52] Handa [57]	Brown 2013 [41] Brown 2017 [42] Brown 2014 [48] Barker 2017 [56] Hadley 2015 [55] Esposito‐Smythers 2017 [43] Houck 2016 [44] Jani 2016 [45] Kendall 2020 [46] Logie 2015 [53] Pearson 2019 [47] Puffer 2016 [50] Thurman 2018 [51] Zellner 2016 [52] Handa [57] Donenberg 2015 [49]
Key Populations (n = 7 unique study samples; n = 7 unique analyses)		Johnson 2015 [64] Newcomb 2017 [67]	Eke 2019 [63] Johnson 2015 [64] Mimiaga 2012 [65] Mimiaga 2019 [66] O'Cleirigh 2019 [68] Tobin 2017 [70]		Eke 2019 [63] Johnson 2015 [64] Mimiaga 2012 [65] O'Cleirigh 2019 [68] Tobin 2017 [70]	Eke 2019 [63] Johnson 2015 [64] Mimiaga 2012 [65] Mimiaga 2019 [66] Newcomb 2017 [67] O'Cleirigh 2019 [68] Tobin 2017 [70]
Integrated Services (n = 1 unique study samples; n = 1 unique analyses)				Systems integration Shaikh 2016 [94]	Shaikh 2016 [94]	Shaikh 2016 [94]

AYW, adolescents and young women; MH, mental health; RCT, randomized controlled trial.

^a^ i.e. emotion regulation, affect management, stress reduction, externalizing, internalizing behaviours

^b^ i.e. depressive symptoms, anxiety, aggressive behaviour, posttraumatic stress symptoms, self‐harm acts, anhedonia.

To our knowledge, this is the first review of global research on mental health and HIV prevention among adolescents and young women, key populations and people with SMI that includes multiple prevention modalities. We aimed to understand how mental disorder symptoms influence risk for HIV and preventive intervention outcomes among three populations vulnerable to HIV infection. Overall, this selection of studies, dominated by behavioural interventions to reduce sexual risk, suggests that poorer mental health is associated with HIV risk behaviour. Study findings help to answer two questions: 1) What do we know about the relationship between mental health and HIV prevention behaviours and 2) What do we know about the interventions that address mental health in the context of HIV prevention?

### 3.7 The relationship between mental health and HIV prevention behaviours

Having symptoms of a mental health condition was more often associated with fewer HIV prevention behaviours. In all but one study, depression impaired PrEP adherence. In the majority of HIV testing and counselling studies, having fewer symptoms of a mental disorder increased HIV testing. Depressive, anxiety and trauma symptoms usually reduced the likelihood of condom use or condom self‐efficacy.

In a subset of studies, depressive symptoms (current or past) were associated with a greater likelihood of getting an HIV test, and with PrEP initiation [60]. In these cases, the negative effect of poor mental health may be mitigated by other factors that facilitate taking action (e.g. high perceived self‐efficacy [60]), or poor mental health may enhance recognition of vulnerability and a need for support [77, 89]. Evidence also showed that learning one’s HIV status, whether negative or positive, did not worsen depressive symptoms or increase suicidality [79].

The relationship between HIV risk and mental health is sometimes indirect. For adolescents, parent–child communication skills, parenting styles and parental mental health status influenced successful sexual communication, which in turn reduced HIV risk [55]. Consistent with this finding, a recent review highlighted the benefits of family strengthening interventions for the mental health of youth affected by or living with HIV [96]. Gender and social adversity add to the complexity of understanding these associations. Cash transfers led to better HIV risk outcomes for boys and girls, but better mental health outcomes for boys [57]. Nevertheless, for vulnerable girls, access to social resources such as free school or cash transfers reduced behaviours directly and indirectly related HIV risk [57, 62]. HIV programme implementers must also consider how the social vulnerabilities of adolescents may increase the risk of coercive participation in HIV prevention or may indirectly create barriers to interventions (e.g. VMMC), and consequently, greater emotional stressors for young people [61].

### 3.8 What do we know about interventions that address mental health in the context of HIV prevention?

Prevention scientists emphasize the importance of combination prevention and comprehensive, layered approaches that address contextual and individual risk factors for HIV prevention [34, 97, 98]. Relatively few examples of such comprehensive approaches emerged in our search. Although some interventions integrated elements to address intrapersonal, community and structural stressors (e.g. self‐esteem, discrimination, minority stress, negative sexual identity, community connectedness, access to care) with HIV prevention [53, 63], these may not be sufficient to reduce symptoms of depression, trauma or severe anxiety. When they yield positive effects on mental health, more research is needed to understand which components and delivery modes facilitate these outcomes. Assessment also influences outcomes: most studies in the sample utilized screening tools to assess mental health status, but did not always distinguish between diagnoses and symptoms. Some studies assessed mental health even if the HIV preventive intervention had no mental health component.

The thirteen studies in Table 5 that reported HIV risk reduction and improved mental health outcomes used three broad intervention approaches. At the systems level, effective linkage of key populations to HIV testing and mental health services occurred through robust referrals within a community setting managed by a trusted community organization and peer network [94]. The individual‐level behavioural interventions, conducted in North America and Africa, applied elements of evidence‐based psychological therapies or psychoeducation within a structured HIV risk reduction intervention for adolescents and young women and for key populations. Across these studies, the sample size, duration and strength of effects, and specification of the mental health component varied considerably. However, several interventions integrating CBT approaches for the treatment of trauma, depression, or self‐harm reduction showed enduring mental health effects nine months to one‐year post‐intervention [43, 68, 70]. Though this latter group of interventions were often delivered by trained mental health specialists in our study sample, current global mental health literature demonstrates that less specialized providers can be trained and supervised to deliver effective, evidence‐based psychological interventions like CBT in community settings [99, 100, 101].

Notably, few new intervention studies for people with SMI have been published since 2012, and studies from Africa, Asia, or Latin America are scarce [2]. The absence in the literature is mirrored by diminishing attention to HIV prevention, access to HIV services in public mental health settings in the United States [95]. Although social functioning—including establishing intimacy and expressing sexuality—is an essential part of the recovery process for people living with SMI, sexual health is largely unaddressed in typical mental health services [24, 102].

### 3.9 Implications for integrating HIV prevention and mental health and achieving global HIV targets

The Global AIDS Targets for 2025 call for «people‐centred and context‐specific integrated approaches» so that 90% of people at high risk of HIV are linked to services for mental health, sexual and gender‐based violence and other relevant care [103]. The results of our review suggest that mental health and HIV prevention could be integrated by (1) identifying community partners and leaders for co‐design, delivery and linkage of HIV and mental health resources and services; (2) using task shifting to train adherence counsellors, peers and nurses to administer evidence‐based psychological therapies (e.g. problem‐solving therapy, CBT, cognitive processing therapy) embedded in theory‐based risk reduction for populations experiencing trauma and depression [104]; (3) making use of available mental health capacity‐building resources from the World Health Organization and other sources [105, 106, 107]; (4) supporting gender‐sensitive structural interventions for young people, like access to free schooling for girls and vulnerable youth; (5) expanding access to family‐based interventions that enhance parenting and communication skills and (6) introducing a mental health component to adherence support for PrEP. True integration requires shared human resources, budgeting, and planning across HIV and mental health services in partnership with diverse community stakeholders [106, 108, 109, 110]. Prevention service providers can learn from task‐sharing interventions that integrate mental health and HIV care [111, 112, 113].

### 3.10 Limitations

Our study has several limitations. The included studies were heterogeneous and precluded the use of a meta‐analysis of the results and effect sizes. Our review was systematized, though not systematic, and we may not have captured all representative studies. We did not rate the quality of the studies or conduct a bias assessment, but reported findings qualitatively. Although we captured studies from a diverse set of countries, the majority of studies are from the United States and interventions reflect the contextual specificities of the study populations and settings. Self‐report of HIV risk behaviours, non‐randomized study designs and small sample sizes may bias some study findings. Despite these weaknesses, the included study results reflect a broad range of countries and support the assertion that poorer mental health is linked to fewer HIV prevention behaviours and activities globally and that integrated interventions can reduce risk.

## 4 Conclusions

Consistent with previous studies, current evidence suggests that mental health conditions are more often associated with poor HIV prevention outcomes, and integrated approaches are urgently needed to address overlapping vulnerabilities among key populations, vulnerable groups and individuals with SMI. Our review contributes a new synthesis of global literature on mental health and HIV prevention, spanning a broad range of prevention modalities; studies from high‐, middle‐ and low‐income countries; and diverse samples of key populations, high‐risk groups, and people with SMI. We highlight the components of interventions that address symptoms of mental illness or psychological processes that influence mental health status. Importantly, these findings, in concert with the broader global mental health literature [11, 114, 115], suggest that integrating structural, social and individual‐level HIV prevention and mental health interventions is feasible in diverse community settings. A renewed focus on implementing these integrated interventions and services could contribute to ending the AIDS epidemic, and specifically, to achieving the 2025 Global AIDS targets.

## Competing interests

The authors have declared no conflict of interest.

## Authors’ contributions

PC, TC and JV developed search criteria. TC, JV and LO completed the primary title/abstract review of studies. PC, TC, JV, LO, LC and JS completed the secondary full‐text review of studies. PC, TC, JV, LO, LC, BW and JS assisted with data coding and analysis. PC, TC, JV, LO, LC, JS and CK contributed to development, writing and editing of this manuscript.

## Abbreviations

ACEs, Adverse childhood experiences; ADS, Affect Dysregulation Scale; AE’s, Adverse events; AGYW, Adolescent girls and young women; AI/AN, American Indian/Alaska Native; BPRS, The Expanded Brief Psychiatric Rating Scale; BSI, Brief Symptom Inventory; CASA, The Child and Adolescent Services Assessment; CDI, Children's Depression Inventory; C‐DISC‐IV, Computerized Diagnostic Interview Schedule for Children; CES‐D, Center for Epidemiological Studies Depression Scale; CES‐D‐R, Center for Epidemiological Studies Depression Scale Revised; CMD, Common mental disorders; DANVA, Diagnostic Analysis of Nonverbal Accuracy; DASS, Depression, Anxiety, and Stress scale; DERS, Difficulties in Emotion Regulation Scale; DISC, Diagnostic Interview Schedule for Children 4.0; DSM‐IV, Diagnostic and Statistical Manual of Mental Disorders, fourth edition; DTS, Davidson Trauma Scale; EMR, Electronic medical record; EPDS, The Edinburgh Postnatal Depression Scale; ER, Emotion regulation; FSW, Female sex worker; GAD, Generalized Anxiety Disorder; GHQ12, General Health Questionnaire; GSI, Global Severity Index; HADS, Hospital Anxiety and Depression Scale; HIV, Human Immunodeficiency Virus; HRQoL SF‐12, Health‐Related Quality of Life SF‐12; HSCL, Hopkins Symptom Checklist; HSCL‐D, Hopkins Symptoms Checklist for Depression; HTC, HIV Testing and Counselling; LBQ, Lesbian Bisexual Queer; MADRS, Montgomery‐Asberg Depression Rating Scale; MASC, Multi‐Dimensional Anxiety Scale for Children; MH, Mental health; MINI PLUS, Mini International Neuropsychiatric Interview – PLUS; MINI, Mini International Neuropsychiatric Interview; MINI‐Kid, Mini International Psychiatric Interview for Children and Adolescents; MSM, Men who have sex with men; NR, Not reported; OVC, Orphans and vulnerable children; PHQ, Patient Health Questionnaire; PrEP, Pre‐Exposure Prophylaxis; PROMIS, Patient‐Reported Outcomes Measurement Information System Depression—Short Form 8b; PTSD, Post‐Traumatic Stress Disorder; PWID, Persons who inject drugs; QIDS, Quick Inventory of Depressive Symptomatology; RCMAS, Revised Children's Manifest Anxiety Scale; RCT, Randomized control trial; SCID, Structured Clinical Interview for DSM‐IV Diagnosis; SCL‐90R, The Symptom Checklist‐90 Revised; SDQ, Strengths and Difficulties Questionnaire; SI, Suicidal intent; SITBI 2.0‐SF, Self‐Injurious Thoughts and Behaviour Interview 2.0 ‐ Short Form; SMI, Serious Mental Illness; SSQ, Shona Symptom Questionnaire; STI, Sexually transmitted infections; SW, Sex worker; TAS, Toronto Alexithymia Scale; TasP, Treatment as prevention; TG, Transgender person; TGW, Transgender women; THQ, Trauma History Questionnaire; UNICEF, United Nations Children's Fund; VMMC, Voluntary medical male circumcision; YSR, Youth Self Report.

## Supporting information


**Additional file S1**. Search strategy.
