# Supplementary material for: Intervening for HIV prevention and mental health: a review of global literature
Source: J Int AIDS Soc. 2021 Jun 24;24(Suppl 2):e25710. doi: 10.1002/jia2.25710 (PMC8222838; doi:10.1002/jia2.25710)
Supplement: Supplementary file 1 — Additional file S1. Search strategy. [file JIA2-24-e25710-s001.docx]

# Additional File 1: Search Strategy

## PubMed (n=1255)

### Adolescents and Young Women (n=560)

((mental health[mh] OR "Mental Disorders"[mh] OR “mental disorder*”[tw] OR “anxiety”[tiab] OR “depression”[tiab] OR “ptsd”[tiab] OR “post-traumatic stress”[tiab]) AND ("HIV Infections/prevention and control"[MAJR] OR "HIV Infections/prevention and control"[mh] OR “hiv prevention”[tiab]) AND (“Intervention*”[tiab] OR “Program*”[tiab] OR “Service*”) AND ((((youth[tiab] OR adolescent[mh] OR adolescen*[tiab] OR "young women"[tiab] OR teenager*[tiab] OR teen*[tiab]) AND (mental health[mh] OR "Mental Disorders"[mh] OR "mental disorder*"[tw] OR "anxiety"[tiab] OR "depression"[tiab] OR "ptsd"[tiab] OR "post-traumatic stress"[tiab])) AND ("HIV Infections/prevention and control"[MAJR] OR "HIV Infections/prevention and control"[mh] OR "hiv prevention"[tiab])) AND ("Intervention*"[tiab] OR "Program*"[tiab] OR "Service*"))

### SMI (n=40)

(("HIV Infections/prevention and control"[MAJR] OR "HIV Infections/prevention and control"[mh] OR “hiv prevention”[tiab]) AND (“Intervention*”[tiab] OR “Program*”[tiab] OR “Service*”) AND ((((Schizophrenia[mh] OR "Paranoid Disorders"[mh] OR "Psychotic Disorders"[mh] OR "Bipolar Disorder"[mh:noexp] OR "Depressive Disorder, Major"[mh] OR "Antipsychotic Agents"[mh] OR "Serious Mental Illness"[tiab] OR "Serious Mental Illnesses"[tiab] OR "Severe Mental Illness"[tiab] OR "Severe Mental Illnesses"[tiab] OR "Seriously Mentally Ill"[tiab] OR "Severely Mentally Ill"[tiab] OR "Serious Mental Disorder"[tiab] OR "Serious Mental Disorders"[tiab] OR "Severe Mental Disorder"[tiab] OR "Severe Mental Disorders[tiab]" OR Schizophrenia[tiab] OR "Paranoid Disorders"[tiab] OR "Psychotic Disorders"[tiab] OR "Bipolar Disorder"[tiab] OR "Depressive Disorder, Major"[tiab] OR Schizophrenias[tiab] OR "Schizophrenic Disorders"[tiab] OR "Schizophrenic Disorder"[tiab] OR "Dementia Praecox"[tiab] OR "Paranoid Disorder"[tiab] OR "Paranoid Psychoses"[tiab] OR "Psychotic Disorder"[tiab] OR "Psychosis"[tiab] OR "Psychoses"[tiab] OR "Schizoaffective Disorder"[tiab] OR "Schizoaffective Disorders"[tiab] OR "Schizophreniform Disorders"[tiab] OR "Schizophreniform Disorder"[tiab] OR "Brief Reactive Psychoses"[tiab] OR "Brief Reactive Psychosis"[tiab] OR "Bipolar Disorders"[tiab] OR "Manic-Depressive Psychosis"[tiab] OR "Manic Depressive Psychosis"[tiab] OR "Bipolar Affective Psychosis"[tiab] OR "Manic-Depressive Psychoses"[tiab] OR Mania[tiab] OR Manias[tiab] OR "Manic State"[tiab] OR "Manic States"[tiab] OR "Bipolar Depression"[tiab] OR "Manic Disorder"[tiab] OR "Manic Disorders"[tiab] OR "Major Depressive Disorders"[tiab] OR "Major Depressive Disorder"[tiab] OR "Involutional Psychoses"[tiab] OR "Involutional Psychosis"[tiab] OR "Involutional Depression"[tiab] OR "Involutional Melancholia"[tiab] OR Antipsychotic[tiab] OR Antipsyhotics[tiab] OR "Major Mental Illness"[tiab] OR "Major Mental Illnesses"[tiab] OR "Major Psychiatric Disorder"[tiab] OR "Major Psychiatric Disorders"[tiab] OR "Serious Psychiatric Disorder"[tiab] OR "Serious Psychiatric Disorders"[tiab] OR "Severe Psychiatric Disorder"[tiab] OR "Severe Psychiatric Disorders"[tiab])) AND ("HIV Infections/prevention and control"[MAJR] OR "HIV Infections/prevention and control"[mh] OR "hiv prevention"[tiab])) AND ("Intervention*"[tiab] OR "program*"[tiab] OR "service*"[tiab]) AND (2010:2020[pdat])))

### Key pop (n=959)

(((mental health[mh] OR "Mental Disorders"[mh] OR “mental disorder*”[tw] OR “anxiety”[tiab] OR “depression”[tiab] OR “ptsd”[tiab] OR “post-traumatic stress”[tiab]) AND ("HIV Infections/prevention and control"[MAJR] OR "HIV Infections/prevention and control"[mh] OR “hiv prevention”[tiab]) AND (“Intervention*”[tiab] OR “Program*”[tiab] OR “Service*”)) AND (((("key population*"[tiab] OR "Homosexuality, Male"[Mesh] OR "MSM"[tiab] OR "men who have sex with men"[tiab] OR "Homosexuality, Male"[mh] OR "PWID" [tiab] OR "people who inject drugs"[tiab] OR "IDU"[tiab] OR "injection drug user*"[tiab] OR "Sex Workers"[Mesh] OR "sex workers"[tiab] OR “sell sex”[tiab] OR “exchange sex”[tiab] OR "Prisoners"[Mesh] OR "prisoners"[tiab] OR "Sexual and Gender Minorities"[Mesh] OR "Transgender Persons"[Mesh] OR "transgender"[tiab] OR "trans men"[tiab] OR "trans women"[tiab]) AND (mental health[mh] OR "Mental Disorders"[mh] OR "mental disorder*"[tw] OR "anxiety"[tiab] OR "depression"[tiab] OR "ptsd"[tiab] OR "post-traumatic stress"[tiab])) AND ("HIV Infections/prevention and control"[MAJR] OR "HIV Infections/prevention and control"[mh] OR "hiv prevention"[tiab])) AND ("Intervention*"[tiab] OR "program*"[tiab] OR "service*"[tiab]) AND (2010:2020[pdat]))

## Web of Science (n=829)

### Adolescents and Young Women (n=377)

TS= ("HIV prevention" OR "HIV education" or (HIV and prevention) ) AND TS= ("mental health" or "mental disorder*" or anxiety or depression or ptsd or "post-traumatic stress" or "adverse childhood experience*") AND TS= (intervention* or program* or service*) AND TS= (youth or adolescent* or teen or teenager or teens or young women or ("young adult" and female) )

*Indexes=SCI-EXPANDED, SSCI, A&HCI, ESCI Timespan=2010-2020*

### SMI (n=93)

TS= ("HIV prevention" OR "HIV education" or (HIV and prevention) ) AND TS= (intervention* or program* or service*) AND TS= (Schizophrenia OR "Paranoid Disorders" OR "Psychotic Disorders" OR "Bipolar Disorder"OR "Depressive Disorder, Major" OR "Antipsychotic Agents" OR "Serious Mental Illness*" OR "Severe Mental Illness*" OR "Seriously Mentally Ill" OR "Severely Mentally Ill" OR "Serious Mental Disorder*" OR "Severe Mental Disorder*" OR Schizophrenia OR "Paranoid Disorder*" OR "Psychotic Disorder*" OR Bipolar* OR "Major Depressive Disorder*" OR Schizophren* OR "Dementia Praecox" OR "Paranoid Disorder" OR "Paranoid Psychoses" OR "Psychosis" OR "Psychoses" OR "Schizoaffective Disorder*" OR "Brief Reactive Psychos*" OR "Manic-Depressive Psychosis" OR "Manic Depressive Psychosis" OR "Manic-Depressive Psychoses" OR Mania* OR "Manic State*" OR "Manic Disorder*" OR "Involutional Psychoses" OR "Involutional Psychosis" OR "Involutional Depression" OR "Involutional Melancholia" OR Antipsychotic* OR "Major Mental Illness*" OR "Major Psychiatric Disorder*" OR "Serious Psychiatric Disorder*" OR "Severe Psychiatric Disorder*")

*Indexes=SCI-EXPANDED, SSCI, A&HCI, ESCI Timespan=2010-2020*

### Key Pop (n=459)

TS= ("HIV prevention" OR "HIV education" or (HIV and prevention) ) AND TS= ("mental health" or "mental disorder*" or anxiety or depression or ptsd or "post-traumatic stress" or "adverse childhood experience*") AND TS= (intervention* or program* or service*) AND TS= (“key population*” OR “MSM” OR “men who have sex with men” OR “Homosexuality” OR "sex worker*" or “sell sex” OR “exchange sex” OR prisoner* OR incarcerate* OR "sexual and gender minorities" OR “transgender” OR “trans men” OR “trans women” OR “PWID” OR “people who inject drugs” OR “IDU” OR “injection drug user*”)

*Indexes=SCI-EXPANDED, SSCI, A&HCI, ESCI Timespan=2010-2020*

## CINAHL (n=201)

### Adolescents and Young Women (n=135)

(("HIV infections/prevention and control" OR MM "HIV Infections+/PC" OR MJ "HIV Infections+/PC" OR TI “HIV prevention” OR AB “HIV prevention”) AND (MH "Mental Health" OR MH "Mental Disorders" OR TX "Mental Disorder" OR TI Anxiety OR AB Anxiety OR TI Depression OR AB Depression OR TI PTSD OR AB PTSD OR TI post-traumatic stress OR AB post-traumatic stress OR MH "Adverse Childhood Experiences") AND LA English AND (TI intervention OR AB intervention OR TI program* OR AB program* OR TI service* OR AB service*) AND (TX youth OR MH adolescent OR TX adolescen* OR TX adolescent OR TX teenager* OR TX teen* OR ((TX "young women" OR TX young adult OR MH "young adult") AND female))

### SMI (n=79)

((("HIV infections/prevention and control" OR MM "HIV Infections+/PC" OR MJ "HIV Infections+/PC" OR TI “HIV prevention” OR AB “HIV prevention”) AND ((MH "Mental Health" OR MH "Mental Disorders" OR TX "Mental Disorder" OR TI Anxiety OR AB Anxiety OR TI Depression OR AB Depression OR TI PTSD OR AB PTSD OR TI post-traumatic stress OR AB post-traumatic stress OR MH "Adverse Childhood Experiences") AND LA English) AND (TI intervention OR AB intervention OR TI program* OR AB program* OR TI service* OR AB service*) AND ((MH Schizophrenia+ OR MH "Paranoid Disorders+" OR MH "Psychotic Disorders+" OR MH "Bipolar Disorder" OR MH Depression OR MH Antipsychotic Agents+" OR TI "Serious Mental Illness" OR AB "Serious Mental Illness" OR TI "Serious Mental Illnesses" OR AB "Serious Mental Illnesses" OR TI "Severe Mental Illness" OR AB "Severe Mental Illness" OR TI "Severe Mental Illnesses" OR AB "Severe Mental Illnesses" OR TI "Seriously Mentally Ill" OR AB "Seriously Mentally Ill" OR TI "Severely Mentally Ill" OR AB "Severely Mentally Ill" OR TI "Serious Mental Disorder" OR AB "Serious Mental Disorder" OR TI "Serious Mental Disorders" OR AB "Serious Mental Disorders" OR TI "Severe Mental Disorder" OR AB "Severe Mental Disorder" OR TI "Severe Mental Disorders" OR AB "Severe Mental Disorders" OR TI Schizophrenia OR AB Schizophrenia OR TI "Paranoid Disorders" OR AB "Paranoid Disorders" OR TI "Psychotic Disorders" OR AB "Psychotic Disorders" OR TI "Bipolar Disorder" OR AB "Bipolar Disorder" OR TI "Depressive Disorder, Major" OR AB "Depressive Disorder, Major" OR TI Schizophrenias OR AB Schizophrenias OR TI "Schizophrenic Disorders" OR AB "Schizophrenic Disorders" OR TI "Schizophrenic Disorder" OR AB "Schizophrenic Disorder" OR TI "Dementia Praecox" OR AB "Dementia Praecox" OR TI "Paranoid Disorder" OR AB "Paranoid Disorder" OR TI "Paranoid Psychoses" OR AB "Paranoid Psychoses" OR TI "Psychotic Disorder" OR AB "Psychotic Disorder" OR TI "Psychosis" OR AB “Psychosis” OR TI "Psychoses" OR AB “Psychoses” OR TI "Schizoaffective Disorder" OR AB "Schizoaffective Disorder" OR TI "Schizoaffective Disorders" OR AB "Schizoaffective Disorders" OR TI "Schizophreniform Disorders" OR AB "Schizophreniform Disorders" OR TI "Schizophreniform Disorder" OR AB "Schizophreniform Disorder" OR TI "Brief Reactive Psychoses" OR AB "Brief Reactive Psychoses" OR TI "Brief Reactive Psychosis" OR AB "Brief Reactive Psychosis" OR TI "Bipolar Disorders" OR AB "Bipolar Disorders" OR TI "Manic-Depressive Psychosis" OR AB "Manic-Depressive Psychosis" OR TI "Manic Depressive Psychosis" OR AB "Manic Depressive Psychosis" OR TI "Bipolar Affective Psychosis" OR AB "Bipolar Affective Psychosis" OR TI "Manic-Depressive Psychoses" OR AB "Manic-Depressive Psychoses" OR TI Mania OR AB Mania OR TI Manias OR AB Manias OR TI "Manic State" OR AB “Manic State” OR TI "Manic States" OR AB “Manic States” OR TI "Bipolar Depression" OR AB “Bipolar Depression” OR TI "Manic Disorder" OR AB “Manic Disorder” OR TI "Manic Disorders" OR AB “Manic Disorders” OR TI "Major Depressive Disorders" OR AB "Major Depressive Disorders" OR TI "Major Depressive Disorder" OR AB "Major Depressive Disorder" OR TI "Involutional Psychoses" OR AB "Involutional Psychoses" OR TI "Involutional Psychosis" OR AB "Involutional Psychosis" OR TI "Involutional Depression" OR AB "Involutional Depression" OR TI "Involutional Melancholia" OR AB "Involutional Melancholia" OR TI Antipsychotic OR AB Antipsychotic OR TI Antipsychotics OR AB Antipsychotics OR TI "Major Mental Illness" OR AB “Major Mental Illness” OR TI "Major Mental Illnesses" OR AB “Major Mental Illnesses” OR TI "Major Psychiatric Disorder" OR AB “Major Psychiatric Disorder” OR TI "Major Psychiatric Disorders" OR AB “Major Psychiatric Disorders” OR TI "Serious Psychiatric Disorder" OR AB “Serious Psychiatric Disoder” OR TI "Serious Psychiatric Disorders" OR “Serious Psychiatric Disorders” OR TI "Severe Psychiatric Disorder" OR AB “Severe Psychiatric Disorder” OR TI "Severe Psychiatric Disorders" OR AB “Severe Psychiatric Disorders”) AND (LA English))

### Key Pop (n=98)

((("HIV infections/prevention and control" OR MM "HIV Infections+/PC" OR MJ "HIV Infections+/PC" OR TI “HIV prevention” OR AB “HIV prevention”) AND ((MH "Mental Health" OR MH "Mental Disorders" OR TX "Mental Disorder" OR TI Anxiety OR AB Anxiety OR TI Depression OR AB Depression OR TI PTSD OR AB PTSD OR TI post-traumatic stress OR AB post-traumatic stress OR MH "Adverse Childhood Experiences") AND LA English) AND (TI intervention OR AB intervention OR TI program* OR AB program* OR TI service* OR AB service*) AND (TI "MSM" OR AB “MSM” OR TI "men who have sex with men" OR AB “men who have sex with men” OR MH "Gay Men" OR TI "PWID" OR AB “PWID” OR TI "people who inject drugs" OR AB “people who inject drugs” OR TI "IDU" OR AB “IDU” OR TI "injection drug user*" OR AB “injection drug user*” OR TI "sex workers" OR AB “sex workers” OR TI “sell sex” OR AB “sell sex” OR TI “exchange sex” OR AB “exchange sex” OR TI prisoner* OR AB prisoner* OR TI incarcerate* OR AB incarcerate* OR TI "prisoners" OR AB “prisoners” OR MH "Prisoners” OR TI "transgender" OR AB “transgender” OR MH "Transgender Persons" OR TI "trans men" OR AB “trans men” OR TI "trans women" OR AB “trans women” OR TI “sexual and gender minorities” or AB “sexual and gender minorities”) AND (LA English)))

## Embase (n=312)

### Adolescents and Young Women (n=174)

('hiv prevention':ti,ab,kw OR 'hiv education':ti,ab,kw OR (hiv:ti,ab,kw AND prevention:ti,ab,kw)) AND ('mental health':ti,ab,kw OR 'mental disorder*':ti,ab,kw OR anxiety:ti,ab,kw OR depression:ti,ab,kw OR ptsd:ti,ab,kw OR 'post-traumatic stress':ti,ab,kw OR 'adverse childhood experience*':ti,ab,kw) AND (intervention*:ti,ab,kw OR program*:ti,ab,kw OR service*:ti,ab,kw) AND (youth:ti,ab,kw OR adolescent*:ti,ab,kw OR teen:ti,ab,kw OR teenager:ti,ab,kw OR teens:ti,ab,kw OR 'young women':ti,ab,kw OR ('young adult':ti,ab,kw AND female:ti,ab,kw)) AND [2010-2020]/py

### SMI (n=61)

('hiv prevention':ti,ab,kw OR 'hiv education':ti,ab,kw OR (hiv:ti,ab,kw AND prevention:ti,ab,kw)) AND (intervention*:ti,ab,kw OR program*:ti,ab,kw OR service*:ti,ab,kw) AND ('paranoid disorders':ti,ab,kw OR 'psychotic disorders':ti,ab,kw OR 'bipolar disorder':ti,ab,kw OR 'depressive disorder, major':ti,ab,kw OR 'antipsychotic agents':ti,ab,kw OR 'serious mental illness*':ti,ab,kw OR 'severe mental illness*':ti,ab,kw OR 'seriously mentally ill':ti,ab,kw OR 'severely mentally ill':ti,ab,kw OR 'serious mental disorder*':ti,ab,kw OR 'severe mental disorder*':ti,ab,kw OR schizophrenia:ti,ab,kw OR 'paranoid disorder*':ti,ab,kw OR 'psychotic disorder*':ti,ab,kw OR bipolar*:ti,ab,kw OR 'major depressive disorder*':ti,ab,kw OR schizophren*:ti,ab,kw OR 'dementia praecox':ti,ab,kw OR 'paranoid disorder':ti,ab,kw OR 'paranoid psychoses':ti,ab,kw OR 'psychosis':ti,ab,kw OR 'psychoses':ti,ab,kw OR 'schizoaffective disorder*':ti,ab,kw OR 'brief reactive psychos*':ti,ab,kw OR 'manic-depressive psychosis':ti,ab,kw OR 'manic depressive psychosis':ti,ab,kw OR 'manic-depressive psychoses':ti,ab,kw OR mania*:ti,ab,kw OR 'manic state*':ti,ab,kw OR 'manic disorder*':ti,ab,kw OR 'involutional psychoses':ti,ab,kw OR 'involutional psychosis':ti,ab,kw OR 'involutional depression':ti,ab,kw OR 'involutional melancholia':ti,ab,kw OR antipsychotic*:ti,ab,kw OR 'major mental illness*':ti,ab,kw OR 'major psychiatric disorder*':ti,ab,kw OR 'serious psychiatric disorder*':ti,ab,kw OR 'severe psychiatric disorder*':ti,ab,kw) AND [2010-2020]/py

### Key Pop (n=98)

('hiv prevention':ti,ab,kw OR 'hiv education':ti,ab,kw OR (hiv:ti,ab,kw AND prevention:ti,ab,kw)) AND (intervention*:ti,ab,kw OR program*:ti,ab,kw OR service*:ti,ab,kw) AND ('mental health':ti,ab,kw OR 'mental disorder*':ti,ab,kw OR anxiety:ti,ab,kw OR depression:ti,ab,kw OR ptsd:ti,ab,kw OR 'post-traumatic stress':ti,ab,kw OR 'adverse childhood experience*':ti,ab,kw) AND (('key population*':ti,ab,kw OR 'msm':ti,ab,kw OR 'men who have sex with men':ti,ab,kw OR 'homosexuality':ti,ab,kw OR 'sex worker*':ti,ab,kw OR 'sell sex':ti,ab,kw OR 'exchange sex':ti,ab,kw OR prisoner*:ti,ab,kw OR incarcerate*:ti,ab,kw OR sexual:ti,ab,kw) AND 'gender minorities':ti,ab,kw OR 'transgender':ti,ab,kw OR 'trans men':ti,ab,kw OR 'trans women':ti,ab,kw OR 'pwid':ti,ab,kw OR 'people who inject drugs':ti,ab,kw OR 'idu':ti,ab,kw OR 'injection drug user*':ti,ab,kw) AND [2010-2020]/py

## PsycInfo (n=165)

### Adolescents and Young Women (n=106)

((MM "AIDS Prevention" OR MJ "AIDS Prevention" OR MA "AIDS Prevention" OR TI "HIV prevention" OR AB "HIV prevention") AND (DE "Mental Health" OR DE "Mental Disorders" OR TX "Mental Disorder" OR TI Anxiety OR AB Anxiety OR TI Depression OR AB Depression OR TI PTSD OR AB PTSD OR TI post-traumatic stress OR AB post-traumatic stress OR DE "Childhood adversity") AND (LA English) AND (TI intervention OR AB intervention OR TI program* OR AB program* OR TI service* OR AB service*) AND (TX youth OR TX adolescen* OR TX adolescent OR TX teenager* OR TX teen* OR ((TX "young women" OR TX young adult) AND female))

### SMI (n=17)

((MM "AIDS Prevention" OR MJ "AIDS Prevention" OR MA "AIDS Prevention" OR TI "HIV prevention" OR AB "HIV prevention") AND (DE "Mental Health" OR DE "Mental Disorders" OR TX "Mental Disorder" OR TI Anxiety OR AB Anxiety OR TI Depression OR AB Depression OR TI PTSD OR AB PTSD OR TI post-traumatic stress OR AB post-traumatic stress OR DE "Childhood adversity") AND (LA English) AND (TI intervention OR AB intervention OR TI program* OR AB program* OR TI service* OR AB service*) AND ((MA Schizophrenia OR MA "Paranoia (Psychosis)" OR MA Psychosis OR MA "Bipolar Disorder" OR MA Major Depression OR MA “Neuroleptic Drugs" OR TI "Serious Mental Illness" OR AB "Serious Mental Illness" OR TI "Serious Mental Illnesses" OR AB "Serious Mental Illnesses" OR TI "Severe Mental Illness" OR AB "Severe Mental Illness" OR TI "Severe Mental Illnesses" OR AB "Severe Mental Illnesses" OR TI "Seriously Mentally Ill" OR AB "Seriously Mentally Ill" OR TI "Severely Mentally Ill" OR AB "Severely Mentally Ill" OR TI "Serious Mental Disorder" OR AB "Serious Mental Disorder" OR TI "Serious Mental Disorders" OR AB "Serious Mental Disorders" OR TI "Severe Mental Disorder" OR AB "Severe Mental Disorder" OR TI "Severe Mental Disorders" OR AB "Severe Mental Disorders" OR TI Schizophrenia OR AB Schizophrenia OR TI "Paranoid Disorders" OR AB "Paranoid Disorders" OR TI "Psychotic Disorders" OR AB "Psychotic Disorders" OR TI "Bipolar Disorder" OR AB "Bipolar Disorder" OR TI "Depressive Disorder, Major" OR AB "Depressive Disorder, Major" OR TI Schizophrenias OR AB Schizophrenias OR TI "Schizophrenic Disorders" OR AB "Schizophrenic Disorders" OR TI "Schizophrenic Disorder" OR AB "Schizophrenic Disorder" OR TI "Dementia Praecox" OR AB "Dementia Praecox" OR TI "Paranoid Disorder" OR AB "Paranoid Disorder" OR TI "Paranoid Psychoses" OR AB "Paranoid Psychoses" OR TI "Psychotic Disorder" OR AB "Psychotic Disorder" OR TI "Psychosis" OR AB “Psychosis” OR TI "Psychoses" OR AB “Psychoses” OR TI "Schizoaffective Disorder" OR AB "Schizoaffective Disorder" OR TI "Schizoaffective Disorders" OR AB "Schizoaffective Disorders" OR TI "Schizophreniform Disorders" OR AB "Schizophreniform Disorders" OR TI "Schizophreniform Disorder" OR AB "Schizophreniform Disorder" OR TI "Brief Reactive Psychoses" OR AB "Brief Reactive Psychoses" OR TI "Brief Reactive Psychosis" OR AB "Brief Reactive Psychosis" OR TI "Bipolar Disorders" OR AB "Bipolar Disorders" OR TI "Manic-Depressive Psychosis" OR AB "Manic-Depressive Psychosis" OR TI "Manic Depressive Psychosis" OR AB "Manic Depressive Psychosis" OR TI "Bipolar Affective Psychosis" OR AB "Bipolar Affective Psychosis" OR TI "Manic-Depressive Psychoses" OR AB "Manic-Depressive Psychoses" OR TI Mania OR AB Mania OR TI Manias OR AB Manias OR TI "Manic State" OR AB “Manic State” OR TI "Manic States" OR AB “Manic States” OR TI "Bipolar Depression" OR AB “Bipolar Depression” OR TI "Manic Disorder" OR AB “Manic Disorder” OR TI "Manic Disorders" OR AB “Manic Disorders” OR TI "Major Depressive Disorders" OR AB "Major Depressive Disorders" OR TI "Major Depressive Disorder" OR AB "Major Depressive Disorder" OR TI "Involutional Psychoses" OR AB "Involutional Psychoses" OR TI "Involutional Psychosis" OR AB "Involutional Psychosis" OR TI "Involutional Depression" OR AB "Involutional Depression" OR TI "Involutional Melancholia" OR AB "Involutional Melancholia" OR TI Antipsychotic OR AB Antipsychotic OR TI Antipsychotics OR AB Antipsychotics OR TI "Major Mental Illness" OR AB “Major Mental Illness” OR TI "Major Mental Illnesses" OR AB “Major Mental Illnesses” OR TI "Major Psychiatric Disorder" OR AB “Major Psychiatric Disorder” OR TI "Major Psychiatric Disorders" OR AB “Major Psychiatric Disorders” OR TI "Serious Psychiatric Disorder" OR AB “Serious Psychiatric Disoder” OR TI "Serious Psychiatric Disorders" OR “Serious Psychiatric Disorders” OR TI "Severe Psychiatric Disorder" OR AB “Severe Psychiatric Disorder” OR TI "Severe Psychiatric Disorders" OR AB “Severe Psychiatric Disorders”) AND (LA English))

### Key Pop (n=84)

((MM "AIDS Prevention" OR MJ "AIDS Prevention" OR MA "AIDS Prevention" OR TI "HIV prevention" OR AB "HIV prevention") AND (DE "Mental Health" OR DE "Mental Disorders" OR TX "Mental Disorder" OR TI Anxiety OR AB Anxiety OR TI Depression OR AB Depression OR TI PTSD OR AB PTSD OR TI post-traumatic stress OR AB post-traumatic stress OR DE "Childhood adversity") AND (LA English) AND (TI intervention OR AB intervention OR TI program* OR AB program* OR TI service* OR AB service*) AND (TI "MSM" OR AB “MSM” OR TI "men who have sex with men" OR AB “men who have sex with men” OR MA "Male Homosexuality" OR TI "PWID" OR AB “PWID” OR TI "people who inject drugs" OR AB “people who inject drugs” OR TI "IDU" OR AB “IDU” OR TI "injection drug user*" OR AB “injection drug user*” OR TI "sex workers" OR AB “sex workers” OR TI “sell sex” OR AB “sell sex” OR TI “exchange sex” OR AB “exchange sex” OR TI prisoner* OR AB prisoner* OR TI incarcerate* OR AB incarcerate* OR TI "prisoners" OR AB “prisoners” OR MA "Prisoners” OR TI "transgender" OR AB “transgender” OR MA "Transgender" OR TI "trans men" OR AB “trans men” OR TI "trans women" OR AB “trans women” OR TI “sexual and gender minorities” or AB “sexual and gender minorities”) AND (LA English)))
